# Reprogramming M2b Macrophages via GPX1 Activation by Selenium Nanoparticles Attenuates Lupus Nephritis

**DOI:** 10.1002/advs.202519981

**Published:** 2025-12-17

**Authors:** Haoran Lv, Guanning Huang, Hongyu Li, Hanzhi Liang, Huajing Peng, Kefei Wu, Wenfang Chen, Dandan Zhang, Kexin Ma, Yufei Du, Siweier Luo, Yi Zhou, Haiping Mao, Wei Chen, Tianfeng Chen, Yiming Zhou, Qinghua Liu

**Affiliations:** ^1^ Department of Nephrology The First Affiliated Hospital Sun Yat‐sen University Guangzhou 518000 China; ^2^ NHC Key Laboratory of Clinical Nephrology (Sun Yat‐sen University) and Guangdong Provincial Key Laboratory of Nephrology Sun Yat‐sen University Guangzhou 518000 China; ^3^ Department of Nephrology Jieyang People's Hospital Jieyang China; ^4^ State Key Laboratory of Bioactive Molecules and Druggability Assessment MOE Key Laboratory of Tumor Molecular Biology Department of Chemistry Jinan University Guangzhou China; ^5^ Department of Pathology The First Affiliated Hospital Sun Yatsen University Guangzhou 518000 China; ^6^ Basic and Translational Medical Research Center Sun Yat‐sen Memorial Hospital Sun Yat‐sen University Guangzhou 518000 China; ^7^ Present address: Department of Nephrology The First Affiliated Hospital Sun Yat‐sen University Guangzhou 518000 China

**Keywords:** lupus nephritis, M2b macrophage, selenium nanoparticles, selenoproteins

## Abstract

Lupus nephritis (LN), a severe complication of systemic lupus erythematosus (SLE), is largely driven by dysregulated macrophage responses. However, the heterogeneity of macrophages hinders the development of targeted therapies for LN. Here, through single‐cell analysis and clinical specimen validation, it is found that pro‐inflammatory M2b macrophages are increased in the kidneys of patients with LN and are strongly associated with clinical indicators. To target and modulate M2b macrophages, mannose‐functionalized selenium nanoparticles are engineered that can selectively suppress M2b polarization and activation by reducing reactive oxygen species (ROS), restoring mitochondrial function, and inducing selenoprotein glutathione peroxidase 1 (GPX1). In vivo, SeZM NPs accumulate in the kidneys of lupus mice and reduce M2b‐derived pro‐inflammatory cytokines, preserving renal structure and function. Together, these findings highlight pro‐inflammatory M2b macrophages as pathogenic drivers of LN and demonstrate the translational potential of selenium‐based nanotherapy.

## Graphical abstract

SeZM NPs selectively target M2b macrophages through mannose–MRC1 (CD206) recognition, enhance GPX1 biosynthesis, and suppress mitochondrial ROS generation, oxidative stress, and JAK–STAT signaling. This dual regulation reduces pro‐inflammatory cytokine release, limits immune cell infiltration and fibrosis, and preserves renal function, providing a macrophage‐subset–specific nanotherapy that addresses the immunopathogenic mechanisms underlying lupus nephritis.

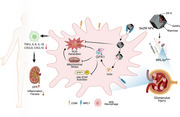

## 1 Introduction

Systemic lupus erythematosus (SLE) is a severe autoimmune disorder that is characterized by immune cell dysregulation and progressive multi‐organ injury.^[^ ^1^ ^]^ Lupus nephritis (LN) is one of the most severe and life‐threatening complications, occurring in ≈50% of patients within five years of diagnosis and frequently progressing to end‐stage renal disease (ESRD).^[^ ^2^ ^]^ LN not only significantly increases the morbidity and mortality of SLE patients but also poses substantial challenges in clinical treatment and management.^[^ ^3^, ^4^ ^]^ Current therapeutics, including corticosteroids, cyclophosphamide, and calcineurin inhibitors, provide non‐specific immunosuppression and disease mitigation, but their use is often limited by systemic toxicity, insufficient long‐term efficacy, and failure to reverse renal damage.^[^ ^5^, ^6^ ^]^ Therefore, there is an urgent need for novel, targeted therapies that could selectively modulate the immune cell response and alleviate LN progression.

Macrophages have been shown to play an important role in the pathogenesis of LN, participating in renal inflammation and fibrosis.^[^ ^7^ ^]^ Notably, renal macrophages exhibit remarkable phenotypic heterogeneity, contributing to distinct phases of tissue injury and repair in LN.^[^ ^8^, ^9^ ^]^ Macrophages are generally grouped into two major subtypes, M1 and M2, where M2 can be further divided into M2a, M2b, and M2c. Recently, M2b macrophages have been getting increased attention for their unique pro‐inflammatory phenotype within the M2 subtypes.^[^ ^10^ ^]^ This subtype of macrophages co‐expresses both M1 and M2 surface markers (CD86 and Mannose Receptor C‐Type 1, MRC1/CD206), and exhibits a strong pro‐inflammatory activity characterized by elevated secretion of cytokines and chemokines (IL‐1β, TNF‐α, CCL2, CXCL9, and other chemokines), which could promote tissue inflammation and fibrosis.^[^ ^11^, ^12^, ^13^ ^]^ However, the role of M2b macrophages in LN and approaches to specifically target them are still poorly understood, partly due to the lack of a selective delivery system capable of cell‐specific immunomodulation.

Nanomedicine offers a promising avenue to address these challenges. Recently, nanoparticles (NPs) have been demonstrated to deliver therapeutic agents to disease‐relevant cells with high spatial and temporal precision.^[^ ^14^ ^]^ Selenium‐based nanoparticles (SeNPs) possess intrinsic anti‐inflammatory, antioxidant, and immunomodulatory properties and have demonstrated efficacy in experimental models of autoimmune and inflammatory diseases.^[^ ^15^, ^16^ ^]^ Despite their pharmacological potential, the application of SeNPs for cell‐specific immunotherapy in LN remains largely unexplored.

Here, we engineered selenium‐based nanoparticles coated with mannose molecules (SeZM NPs) that could selectively target M2b macrophages in lupus nephritis. These nanoparticles effectively modulate M2b macrophage polarization and activation by enhancing the expression of selenoprotein GPx1, reducing reactive oxygen species (ROS) production, and improving mitochondrial function. In vivo, SeZM NPs accumulated in the kidneys of lupus mice and reduced M2b‐derived pro‐inflammatory cytokines, preserving renal structure and function (Table of Contents).

## 2 Results

### 2.1 Single‐Cell Analysis Uncovers M2b Macrophages as a Tractable Target in Lupus Nephritis

**Figure 1:**
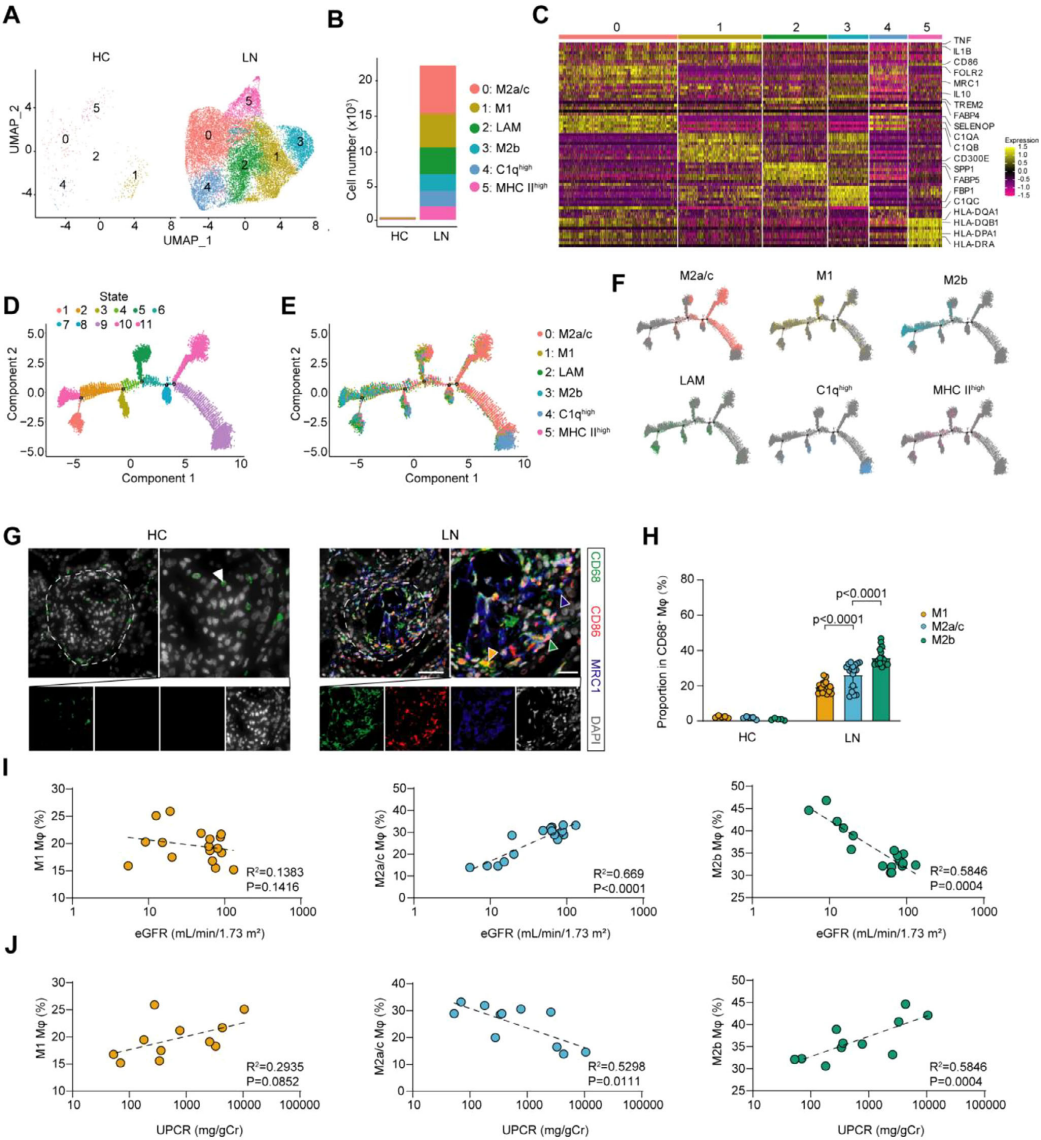
scRNA‐seq identifies pathogenic macrophage subset linked to disease severity in lupus nephritis (LN). A) UMAP of macrophage subsets (Cluster 0–5) from kidneys of healthy controls (HC) and patients with LN. HC n = 6; LN n = 40. B) Average cell number per sample of each macrophage subset in HC and LN groups. C) Heatmap showing representative signature genes of each macrophage cluster. D) Pseudotime trajectory of macrophages colored by developmental state. E,F) Pseudotime trajectory of macrophages colored by cluster identity and distribution along the trajectory. G) Representative fluorescent images of CD68, CD86, and MRC1 (CD206) in the kidneys of HC (n=5) and patients with LN (n=17). White, orange, blue, and green arrowheads indicate M0 (CD68^+^CD86^−^MRC1^−^), M1 (CD68^+^CD86^+^MRC1^−^), M2a/c (CD68^+^CD86^−^MRC1^+^), and M2b (CD68^+^CD86^+^MRC1^+^) macrophages, respectively. Scale bar, 50 µm; scale bar of magnified area, 20 µm. H) Quantification of M1, M2a/c, and M2b macrophages in the kidneys of HC and patients with LN from (G). I,J) Correlation analysis between the M1, M2a/c, and M2b macrophage abundance and clinical indicators of renal functions, including eGFR (I) and UPCR (J).

To explore the macrophage heterogeneity in lupus nephritis (LN), we analyzed the macrophage subset using our previously published single‐cell RNA sequencing (scRNA‐seq) dataset comprising renal biopsies from 40 patients with LN and 6 healthy controls (HCs).^[^ ^17^ ^]^ Unsupervised clustering identified six distinct macrophage subpopulations, including Cluster 0: M2a/c macrophages; Cluster 1: M1 macrophages; Cluster 2: Lipid‐associated macrophages, LAMs; Cluster 3: M2b macrophages; Cluster 4: C1q^high^ tissue‐resident macrophages; and Cluster 5: MHC‐II^high^ macrophages (**Figure** **1**A). Compared to HC samples, LN kidneys exhibited a pronounced increase of all macrophage clusters, with cluster 0 (M2a/c macrophages) as the predominant subset (Figure 1B). Heatmap of signature genes in each macrophage subset revealed specific gene expression patterns: M2a/c was marked by high expression of *FLOR2*, *MRC1*; M1 by *TNF*, *IL1B*, *CD300E*; LAMs by *TREM2*, *SPP1*, *FABP4/5*; M2b by *IL1B*, *MRC1*, *IL10*; C1q^high^ tissue‐resident macrophages by *C1qa*, *C1qb*, *C1qc*; and MHC‐II^high^ macrophages by class II MHC genes (Figure 1C). Interestingly, the heatmap revealed lower expression of the selenium‐associated gene *SELENOP* in the M2b subset compared to M2a/c, implicating selenium‐associated gene as a potential regulator of M2 macrophage polarization. Pseudotime trajectory analysis revealed that M2a/c (Cluster 0), M1 (Cluster 1), and M2b (Cluster 3) macrophages occupy central nodes along the differentiation continuum. Of note, the close proximity of M1 (Cluster 1) and M2b (Cluster 3) in the trajectory suggests that M2b macrophages may functionally resemble M1 and contribute to pro‐inflammatory responses in LN (Figure 1D–F). These results highlight the strong heterogeneity and plasticity of renal macrophages and reveal a previously unrecognized association between M2b macrophages and lupus nephritis.

To validate these transcriptomic findings, we performed multiplex immunofluorescence staining on renal biopsy sections obtained from patients with LN and HCs. Consistent with the scRNA‐seq results, M1 (CD86^+^MRC1^−^), M2a/c (CD86^−^MRC1^+^), and M2b macrophages (CD86^+^MRC1^+^) were markedly enriched in LN kidneys compared to HC samples, where their presence was negligible (Figure 1G). Different from scRNA‐seq results, quantitative analysis of the staining results showed that M2b macrophages are the most abundant macrophage subset in LN kidneys, outnumbering M1 and M2a/c subsets (Figure 1H). Interestingly, the intrarenal abundance of M2a/c and M2b macrophages strongly correlated with established clinical indicators of renal dysfunction, whereas M1 macrophages showed no significant association. Notably, while higher M2a/c macrophage abundance showed a beneficial association with clinical indicators (higher eGFR and lower UPCR), M2b macrophage abundance strongly correlated with worse renal function (lower eGFR and higher UPCR) in LN (Figure 1I,J). These findings suggest that M2b macrophages may exert a unique pathogenic role in LN compared to M1 and M2a/c subsets. Their pronounced accumulation, unique transcriptomic profile, and strong association with clinical indicators of renal dysfunction underscore their potential as a tractable and selective therapeutic target for LN.

### 2.2 Engineering and Physicochemical Characterization of the Immune‐Regulatory Selenium Nanodrugs Targeting Pathogenic M2b Macrophages

**Figure 2:**
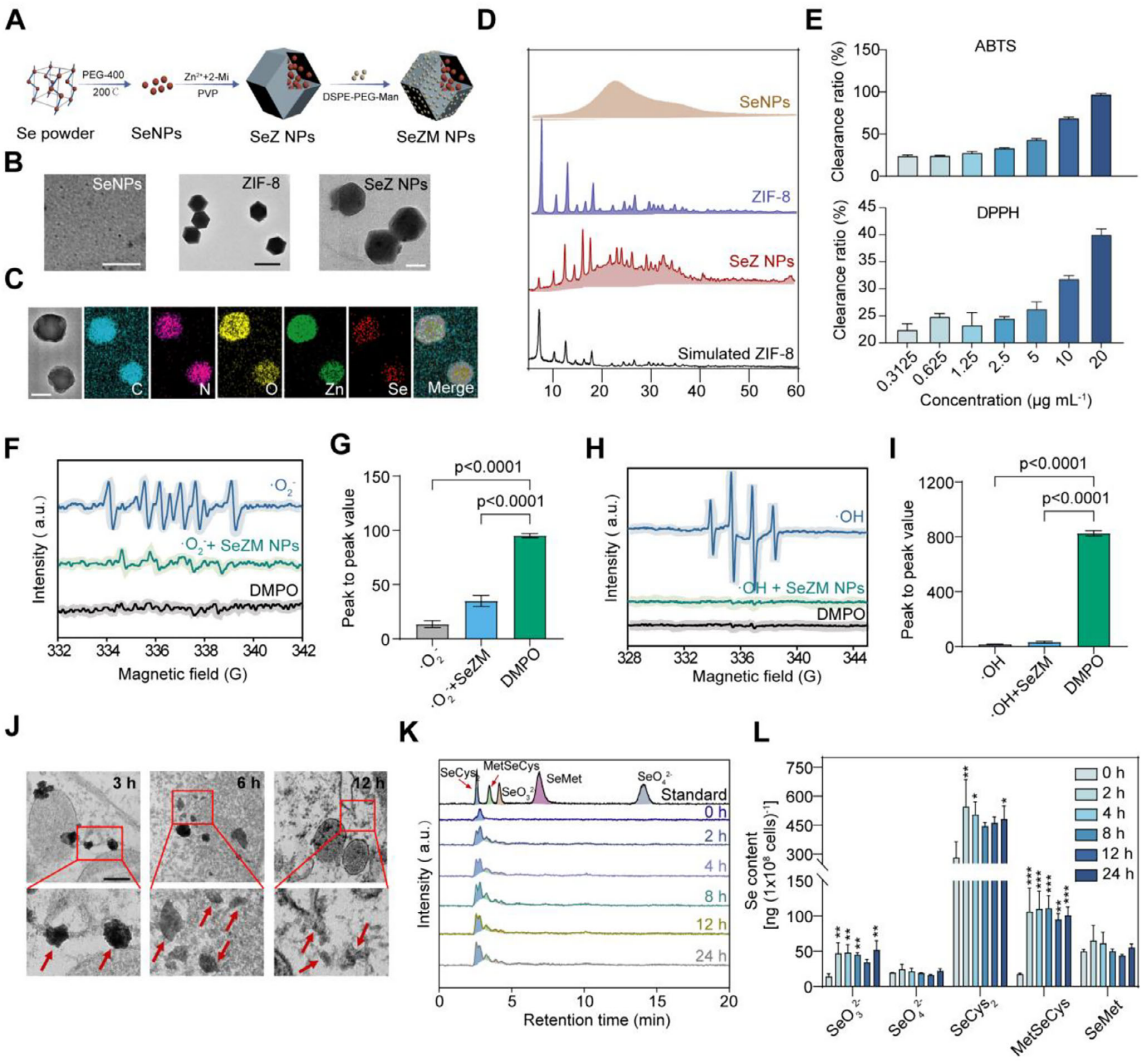
Engineering and multi‐scale characterization of SeZM NPs. A) Schematic graph showing the construction of SeZM NPs. SeNPs: selenium nanoparticles; ZIF‐8 NPs: zeolitic imidazolate framework‐8; SeZ NPs: SeNPs in ZIF‐8 NPs. B) Transmission electron microscopy (TEM) images of SeNPs (scale bar: 100 nm), ZIF‐8 NPs (scale bar: 500 nm), and SeZ NPs (scale bar: 100 nm). C) Elemental mapping of C, N, O, Zn, and Se in SeZ NPs (Scale bar: 200 nm). D) X‐ray diffraction (XRD) patterns of SeNPs, ZIF‐8 NPs, SeZ NPs, and simulated ZIF‐8. E) Evaluation of free radical scavenging capacity of SeZM NPs at different concentrations using ABTS and DPPH assays. F–I) EPR spectra showing the ·O_2−_ (F) and ·OH (H) radical scavenging capacity of SeZM NPs (40 µg mL^−1^), with quantification of peak‐to‐peak values (G,I). J) Representative transmission electron microscopy (TEM) images showing time‐dependent intracellular internalization of SeZM NPs in macrophages at 3, 6, and 12 h post‐treatment. Scale bar: 200 nm. Red arrows indicate SeZM NPs. K,L) HPLC‐ICP‐MS analysis of selenium metabolites (SeO_3_^2−^, SeO_4_^2−^, SeCys_2_, MetSeCys, SeMet) in macrophages treated with SeZM NPs at indicated timepoints (K), with quantification of Se species (L). Data are presented as mean ± s.e.m from n = 3 biologically independent samples; ^*^ *p* < 0.05, ^**^ *p* < 0.01, ^***^ *p* < 0.001 vs 0 h.

Single‐cell RNA sequencing revealed that *SELENOP*, a selenium‐associated gene, is selectively downregulated in M2b macrophages compared to M2a/c, indicating impaired selenium signaling in M2b macrophages. To selectively target and modulate pro‐inflammatory M2b macrophages in lupus nephritis, we designed and engineered mannose‐functionalized selenium nanoparticles embedded in a zeolitic imidazolate framework coated with mannose molecules for specific targeting of M2b macrophages via MRC1 (CD206) (**Figure** **2**A). Ultrasmall selenium nanoparticles (SeNPs; ≈10 ± 2 nm), synthesized via a PEG400‐assisted solvothermal method, were encapsulated within zeolitic imidazolate framework‐8 (ZIF‐8; ≈340 ± 20 nm) to form SeZ nanoparticles (SeZ NPs; ≈180 ± 10 nm) (Figure 2B). Dynamic light scattering (DLS) analysis confirmed the average diameters of SeNPs, ZIF‐8, and SeZ NPs were 410, 457, and 337 nm, respectively (Figure S1A, Supporting Information). The corresponding zeta potentials were −15.8, 19.8, −7.8, and 5.28 mV for Se NPs, ZIF‐8, SeZ NPs, and SeZM NPs, respectively. Mannose conjugation shifted the zeta potential of SeZ NPs from −7.8 to 5.3 mV, potentially enhancing systemic circulation and kidney targeting efficiency (Figure S1B, Supporting Information). Elemental mapping confirmed the uniform integration of selenium within the ZIF‐8 (Figure 1C). X‐ray diffraction (XRD) identified characteristic peaks consistent with the presence of carbon dots and structural integrity of ZIF‐8 (Figure 1D).

X‐ray photoelectron spectroscopy (XPS) was employed to investigate structural modifications in ZIF‐8 following Se NPs incorporation. The O1s spectrum highlighted three oxygen states, with oxygen vacancies (O_b_) linked to ROS scavenging. Doping of SeNPs increased O_b_ content in ZIF‐8 from 24% to 43%, enhancing antioxidant activity (Figure S1C–F, Supporting Information). Se3d spectra demonstrated characteristic Se peaks, including Se3d_3/2_ and Se3d_5/2_, and disappearance of the Se─O peak in SeZ NPs, suggesting successful encapsulation and oxidation prevention (Figure S1G–H, Supporting Information). Other elemental spectra (C1s, Zn2p, N1s) of SeNPs, ZIF‐8, and SeZ NPs showed no novel peaks (Figure S2A–C, Supporting Information).

Excessive production of reactive oxygen species (ROS) by macrophages amplifies the release of pro‐inflammatory cytokines and chemokines in LN, thereby exacerbating renal inflammation and injury.^[^ ^18^, ^19^ ^]^ To evaluate the antioxidant capacity of SeZM NPs, we conducted ABTS and DPPH radical scavenging assays. SeZM NPs exhibited dose‐dependent clearance of both ABTS and DPPH radicals, with scavenging efficiencies reaching ≈44% and ≈40%, respectively, at 5–20 µg mL^−1^ (Figure 2E). To evaluate the impact of SeZM NPs on endogenous reactive oxygen species (ROS), we performed electron paramagnetic resonance (EPR) spectroscopy to assess their scavenging activity against superoxide anions (·O_2−_) and hydroxyl radicals (·OH). As expected, treatment with SeZM NPs led to a marked reduction in EPR signal intensity compared to the untreated group (Figure 2F–I). These results confirm the successful construction of a structurally stable, mannose‐functionalized selenium nanoparticle with well‐defined physicochemical properties, enabling M2b‐specific targeting and immune modulation.

### 2.3 Intracellular Metabolism and Modulation of SeZM NPs in Macrophages

Previous investigations have shown that ZIF metal framework materials are frequently utilized as drug carriers due to their pH‐responsive, sustained‐release properties in mildly acidic environments.^[^ ^20^ ^]^ Nanodrugs internalized by macrophages typically undergo endo‐lysosomal trafficking, followed by controlled degradation in acidic compartments, enabling cargo release and potential therapeutic modulation.^[^ ^21^, ^22^ ^]^ Their metabolic fate depends on nanoparticle composition, cellular redox state, and the expression of degradative enzymes.^[^ ^23^ ^]^ Therefore, we investigated the intracellular metabolism of SeZM NPs in macrophages using transmission electron microscopy (TEM). The TEM images showed that SeZM NPs exhibited gradual size reduction within macrophages over a 12 h period, indicating a slow degradation process (Figure 2J), enabling the gradual release of selenium and subsequent immunomodulation of macrophage function.

Selenocysteine, the 21st amino acid, is incorporated into selenoproteins and plays a crucial role in regulating the redox balance of the matrix.^[^ ^24^, ^25^ ^]^ High‐performance liquid chromatography coupled with inductively coupled plasma mass spectrometry (HPLC‐ICP‐MS) identified intracellular selenium metabolites (SeO_3_^2−^, SeO_4_^2−^, SeCys_2_, MetSeCys, and SeMet), with SeCys_2_ concentrations significantly elevated, suggesting enhanced selenoprotein synthesis via SeCys_2_ production (Figure 2K,L). These results suggest that SeZM NPs might promote the expression of selenoproteins mainly through conversion to the seleno‐amino acid SeCys_2_.

### 2.4 Evaluation of the Biocompatibility and Safety of SeZM NPs

Although selenium exhibits potent antioxidant and immunomodulatory properties, its clinical use has been limited by the narrow therapeutic window and systemic toxicity of conventional formulations.^[^ ^26^, ^27^ ^]^ High doses of inorganic or organic selenium often cause hepatic, renal, or cardiovascular toxicity. To explore the biocompatibility of SeZM NPs, we performed a hemolysis assay. The results showed no morphological changes in erythrocytes at concentrations up to 80 µg mL^−1^, supporting their favorable blood compatibility (Figure S3A–C, Supporting Information). We then evaluated the cytocompatibility in bone marrow‐derived macrophages (BMDMs). Treatment of SeZM NPs up to 30 µg mL^−1^ showed no cytotoxicity or apoptosis across CCK8, live/dead staining, and Annexin V/7‐AAD assays (Figure S3D–H, Supporting Information). Notably, this safe dose exceeds the tolerable limit of traditional selenium compounds by over 1000 fold.^[^ ^24^ ^]^ In vivo, C57BL/6 mice administered a high concentration (10 mg kg^−1^) of SeZM NPs intravenously showed no mortality, behavioral changes, or histological changes of vital organs (Figure S4A, Supporting Information). In addition, serological results for hepatic, renal, and cardiac functions remained unaffected after the treatment (Figure S4B, Supporting Information). These results highlight the favorable biocompatibility and safety of SeZM NPs, supporting their translational potential for lupus nephritis.

### 2.5 SeZM NPs Exhibit Selective Targeting Capability Toward M2b Macrophages

To assess the targeting specificity of SeZM NPs, we first evaluated the uptake levels across different macrophage subpopulations in vitro. BMDMs were polarized into M0, M1, M2a, M2c, and M2b subpopulations, followed by incubation with Rhodamine B‐labeled SeZ or SeZM NPs. Fluorescent microscopy revealed preferential accumulation of SeZM NPs in M2b macrophages, with markedly higher uptake than in other subsets or with selenium‐free NPs (Figure S5A, Supporting Information). Quantitative analysis confirmed significantly increased internalization of SeZM NPs by M2b macrophages (Figure S5B, Supporting Information), suggesting enhanced targeting likely mediated by mannose receptor MRC1 (CD206) interaction. Moreover, qPCR analysis showed that SeZM NPs did not alter the polarization markers or inflammatory genes in M1, M2a, or M2c macrophages, except *Tnf* and *Il1b* in M1 (Figure S5C–E, Supporting Information), demonstrating the specific targeting and modulation effects of SeZM NPs on M2b macrophages.

We next evaluated the M2b macrophage‐targeting capability of SeZM NPs in MRL/lpr mice. Rhodamine B‐labeled SeZ or SeZM NPs were administered intravenously to MRL/lpr mice, and the Rhodamine B‐positive immune cells in splenocytes and PBMCs were analyzed via flow cytometry (Figure S6A, Supporting Information). SeZM NPs showed minimal uptake by T, B, and NK cells, but were robustly internalized by CD11b⁺ macrophages (Figure S6B–F, Supporting Information). Notably, within the Rhodamine B positive macrophage populations, M2b macrophages were markedly enriched following SeZM NPs treatment, accounting for ≈64.9% of targeted macrophages, compared to ≈31.8% in the SeZ group (Figure S6G, Supporting Information). Together, these findings demonstrate that SeZM NPs exhibit strong targeting capability for M2b macrophages both in vitro and in vivo, supporting their application in M2b‐targeted immunomodulatory therapy for lupus nephritis.

### 2.6 Regulation of M2b Macrophage Polarization and Activation by SeZM NPs

**Figure 3:**
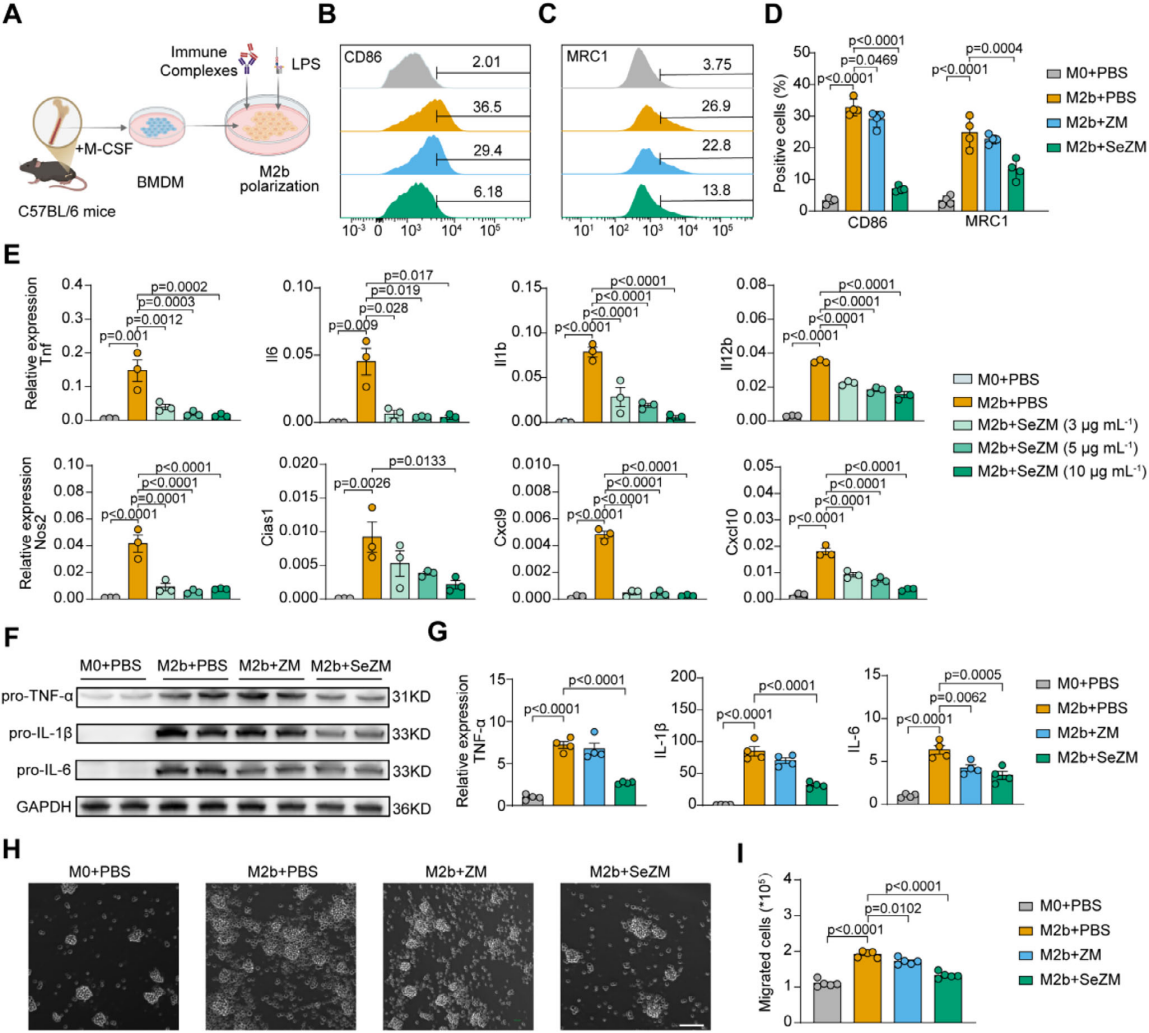
SeZM NPs suppress M2b macrophage polarization and activation in vitro. A) Schematic graph of in vitro polarization of M2b macrophage from bone marrow‐derived macrophages (BMDMs) using immune complex (IC) and lipopolysaccharide (LPS). B–D) Flow cytometry histograms and quantification of CD86 and MRC1 (CD206) expression in M0 and M2b macrophages treated with PBS, selenium‐free ZM NPs (10 µg mL^−1^), or SeZM NPs (10 µg mL^−1^) for 12 h. n = 4 per group. E) qRT‐PCR analysis of pro‐inflammatory cytokines and chemokines in M0 and M2b macrophages treated with PBS or SeZM NPs (3, 5, and 10 µg mL^−1^) for 12 h. n = 3 per group. F,G) Western blotting and densitometric quantification of TNF‐α, IL‐1β, and IL‐6 expression in M0 and M2b macrophages treated with PBS, ZM NPs, or SeZM NPs (10 µg mL^−1^) for 6 h. Cells were treated with Brefeldin A. n = 4 per group. H,I) Representative images (H) and quantification results (I) of CD4^+^ T cell chemotaxis by the supernatants of M2b macrophages treated with PBS and SeZM NPs for 24 h. Scale bar: 100 µm. n = 4 per group.

To examine the regulatory effects of SeZM NPs on M2b polarization, BMDMs were stimulated with lipopolysaccharide (LPS) and immune complexes to induce M2b differentiation (**Figure** **3**A). This induction led to the upregulation of both CD86 and MRC1 (CD206), characteristic markers of M2b macrophages. Treatment with SeZM NPs significantly downregulated both protein expression, whereas selenium‐free ZM NPs had no obvious effect (Figure 3B–D), suggesting SeZM NPs could inhibit M2b polarization.

In addition, SeZM NPs dose‐dependently downregulated multiple pro‐inflammatory genes (*Tnf, Il6, Il1b, Il12b, Nos2, Cias1*) and chemokines (*Cxcl9, Cxcl10*) in M2b macrophages (Figure 3E). At the protein level, SeZM NPs, but not ZM NPs, significantly reduced the levels of tumor necrosis factor‐α (TNF‐α), interleukin‐1β (IL‐1β), and interleukin‐6 (IL‐6) (Figure 3F,G), indicating a potent immunomodulatory effect on M2b polarization and activation. Given that M2b macrophages could promote lupus nephritis progression by recruiting immune cells via various chemokines,^[^ ^24^ ^]^ we next evaluated the effect of SeZM NPs on the T cell chemotaxis of M2b macrophages. Consistent with their elevated expression of Cxcl9 and Cxcl10, M2b macrophages enhanced CD4⁺ T cell chemotaxis, which was markedly attenuated by SeZM NPs but not by ZM NPs (Figure 3H,I). Together, these findings demonstrate that SeZM NPs inhibit M2b macrophage polarization, inflammatory activation, and CD4⁺ T cell recruitment through MRC1‐mediated uptake, highlighting their potential as a targeted immunomodulatory therapy for lupus nephritis.

### 2.7 SeZM NPs Alleviate Oxidative Stress and Restore Mitochondrial Functions in M2b Macrophages

**Figure 4:**
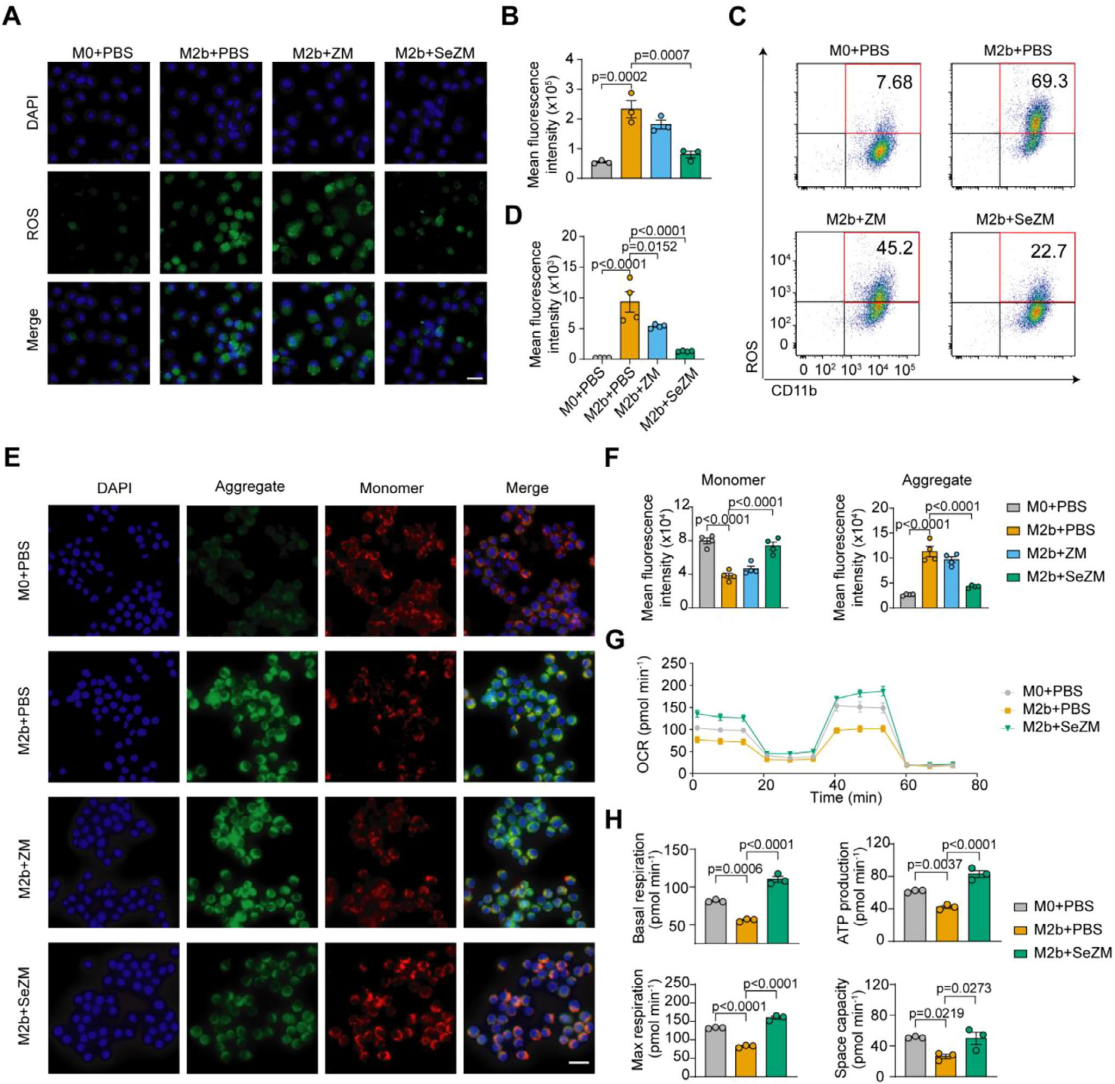
SeZM NPs alleviate oxidative stress and restore mitochondrial functions in M2b macrophages. A,B) Representative fluorescent images (A) and quantification results (B) of intracellular reactive oxygen species (ROS) in M0 and M2b macrophages treated with PBS, ZM NPs (10 µg mL^−1^), or SeZM NPs (10 µg mL^−1^) for 12 h. ROS was stained with DCFH‐DA (green), and nuclei were counterstained with DAPI (blue). Scale bar, 10 µm. n = 3 per group. C,D) Flow cytometry plots (C) and quantification (D) of ROS levels in M0 and M2b macrophages after the indicated treatments for 12 h. n = 4 per group. E) Representative JC‐1 staining images of M0 and M2b macrophages treated with PBS, ZM NPs (10 µg mL^−1^), or SeZM NPs (10 µg mL^−1^) for 6 h. Red fluorescence indicates JC‐1 aggregates (high mitochondrial membrane potential), and green fluorescence indicates monomers (low membrane potential). Scale bar: 10 µm. n = 4 per group. F) Quantification of JC‐1 fluorescence intensities, reflecting the ratio of monomer and aggregate signals as indicators of mitochondrial membrane potential. n = 4 per group. G) Oxygen consumption rate (OCR) profiles of M0 and M2b macrophages treated with PBS or SeZM NPs (10 µg mL^−1^) using a Seahorse XF assay. H) Quantitative analysis of mitochondrial respiratory parameters derived from (G), including basal respiration, ATP production, maximal respiration, and spare respiratory capacity. n = 3 per group.

ROS play a pivotal role in macrophage polarization and activation, and their excessive production is closely linked to autoimmunity.^[^ ^28^ ^]^ To investigate the antioxidative potential of SeZM NPs in macrophages, we assessed intracellular ROS levels using DCFH‐DA staining. Compared to M0 macrophages, M2b macrophages exhibited elevated ROS levels, which were significantly reduced upon treatment with SeZM NPs, but not with selenium‐free ZM NPs (**Figure** **4**A,B). This ROS‐scavenging effect was further confirmed by flow cytometry (Figure 4C,D), suggesting that SeZM NPs could suppress M2b activation by attenuating oxidative stress.

Given the central role of mitochondria in ROS production,^[^ ^29^ ^]^ we assessed mitochondrial function in M2b macrophages. Mitochondrial membrane potential (MMP), a key indicator of mitochondrial health, was measured using JC‐1 dye. M2b macrophages exhibited reduced MMP, reflected by increased green (monomeric) and decreased red (aggregated) JC‐1 fluorescence, indicating mitochondrial dysfunction. Interestingly, SeZM NPs, but not ZM NPs, effectively restored MMP (Figure 4E,F). We further evaluated mitochondrial respiration and glycolysis via the Seahorse assay. Compared to M0 macrophages, M2b macrophages showed impaired mitochondrial respiration levels, characterized by reduced basal respiration, ATP production, maximal respiration, and spare capacity. Notably, treatment with SeZM NPs reversed these deficiencies (Figure 4G,H), indicating improved mitochondrial function by SeZM NPs in M2b macrophages. Together, these findings demonstrate that SeZM NPs regulate M2b macrophages by suppressing ROS accumulation and restoring mitochondrial function in these cells.

### 2.8 SeZM NPs Regulate the JAK‐STAT Signaling of M2b Macrophages via Selenoprotein GPx1

**Figure 5:**
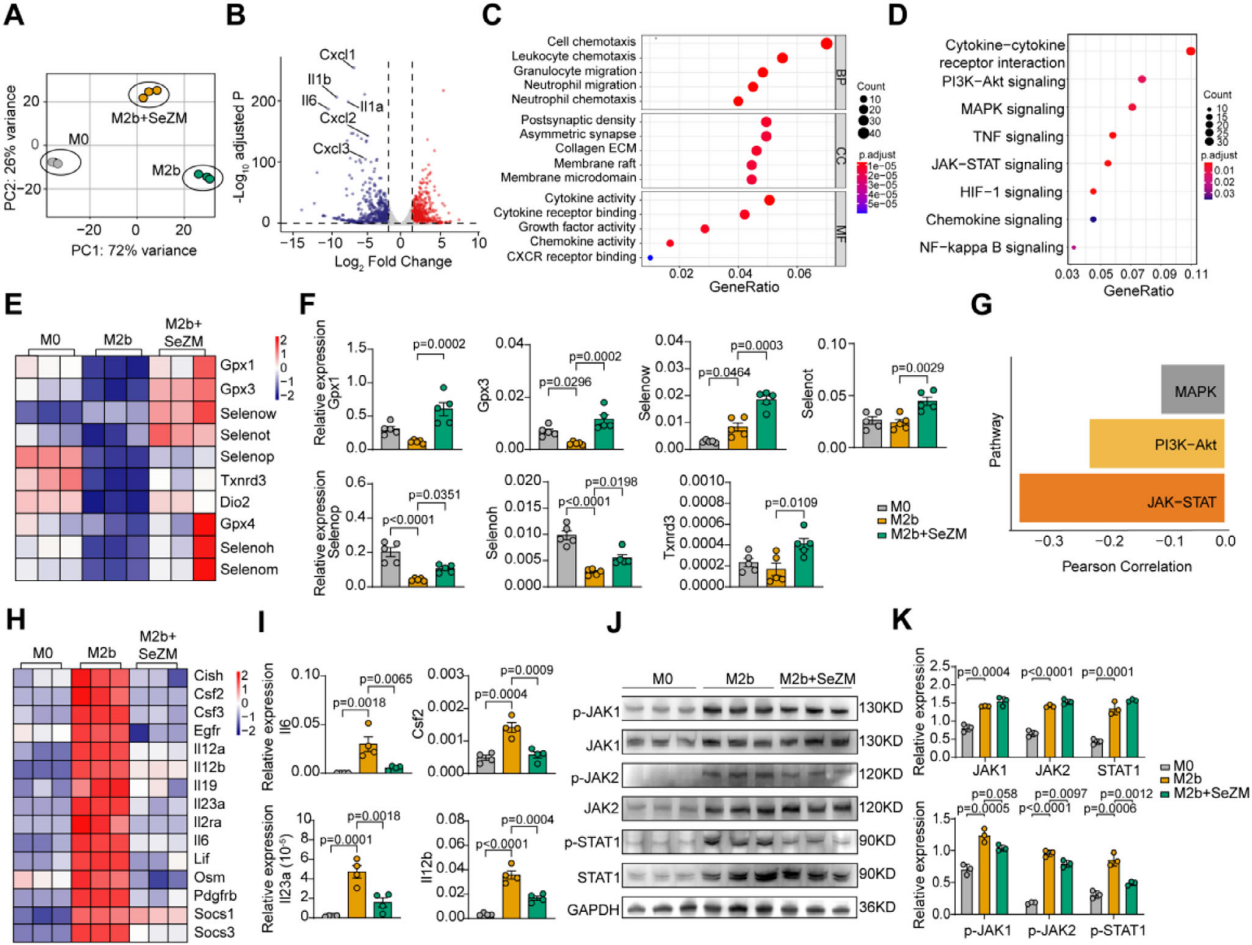
Transcriptomic analysis elucidates the immunomodulatory mechanism of SeZM NPs on M2b macrophages. A) PCA of RNA‐seq data from M0 and M2b macrophages treated with PBS or SeZM NPs (10 µg mL^−1^) for 12 h. n = 3 biological replicates per group. B) Volcano plot of differentially expressed genes (DEGs) between M2b and M2b+SeZM groups. C) Gene Ontology (GO) enrichment analysis of downregulated DEGs in M2b+SeZM group. D) KEGG pathway enrichment of downregulated DEGs showing attenuation of inflammatory and metabolic signaling, including JAK‐STAT, MAPK, and PI3K‐Akt pathways. E) Heatmap of differentially expressed selenoprotein genes among M0, M2b, and M2b+SeZM groups. F) RT‐PCR examination of the mRNA relative expression of differentially expressed selenoproteins. n = 5 per group. G) Pearson correlation analysis between Gpx1 expression and activity scores of enriched signaling pathways from (D). H) Heatmap of the downregulated DEGs in the JAK‐STAT pathway among three groups. I) RT‐PCR examination of the mRNA relative expression of representative genes of the JAK‐STAT pathway. n = 4 per group. J‐K) Western blotting (J) and quantification results (K) of JAK1, phospho‐JAK1, JAK2, phospho‐JAK2, STAT1, and phospho‐STAT1 among three groups. n = 3 per group.

To explore the detailed mechanism of SeZM NPs in the regulation of M2b macrophage polarization and activation, RNA‐sequencing (RNA‐seq) analysis was performed in M0 macrophages treated with PBS (control), M2b macrophages treated with PBS (M2b) and SeZM NPs (M2b+SeZM). Principal component analysis (PCA) revealed clear transcriptomic separation between the three groups, with high intra‐group consistency (**Figure** **5**A). Differentially expressed gene analysis identified 698 upregulated and 720 downregulated genes in the M2b+SeZM group compared to the control group (Figure 5B), including reduced expression of pro‐inflammatory mediators such as *Il1b, Il6, Cxcl1, and Cxcl2*. Gene ontology (GO) and Kyoto Encyclopedia of Genes and Genomes (KEGG) pathway enrichment of downregulated genes revealed significant suppression of immune‐related pathways, including cytokine signaling, TNF signaling, and notably the JAK‐STAT pathway (Figure 5C,D).

Studies have demonstrated that the physiological impacts of selenium are mostly influenced by selenoproteins, which are predominantly composed of the selenium‐containing amino acid, such as selenocysteine, which function as oxidoreductase in cells.^[^ ^30^, ^31^ ^]^ Interestingly, SeZM NPs treatment significantly restored mRNA expression of several selenium‐containing proteins in M2b macrophages, with the *Gpx1* being the most upregulated (Figure 5E), which was further confirmed at the mRNA level (Figure 5F). To assess the functional relevance of *Gpx1*, we conducted pathway correlation analysis, which showed a strong negative association between *Gpx1* expression and activation of the JAK‐STAT pathway, followed by PI3K‐AKT and MAPK signaling (Figure 5G). Indeed, a subset of well‐established JAK‐STAT pathway‐associated pro‐inflammatory genes, *Il6, Csf2, Il23*, and *Il12b*, were found to be suppressed by SeZM NPs in M2b macrophages (Figure 5H). The decreases in mRNA expression of these genes were further confirmed by RT‐PCR in three groups (Figure 5I). As expected, the protein phosphorylation levels of JAK1, JAK2, and STAT1 were significantly upregulated in M2b macrophages. More importantly, treatment with SeZM NPs significantly reduced the phosphorylation levels of JAK2 and STAT1 (Figure 5J,K), indicating that SeZM NPs may regulate the polarization and activation of M2b macrophages via the JAK‐STAT pathway by regulation of selenoprotein GPX1. Taken together, these results elucidate the mechanistic basis underlying the immunomodulatory effects of SeZM NPs on M2b macrophages.

### 2.9 Biodistribution of SeZM NPs in Lupus Nephritis Mice

To assess the biodistribution and targeting specificity in vivo, we generated Indocyanine Green (ICG)‐labeled SeZM and SeZ NPs and injected them intravenously into MRL/lpr lupus mice. Fluorescence imaging revealed that both nanoparticles initially accumulated in the dorsal region post‐injection, but only SeZM NPs exhibited prominent and sustained fluorescence in the kidney region up to 12 h (Figure S7A, Supporting Information). In contrast, SeZ NPs showed rapid clearance from the kidney and retention in the liver. Quantitative analysis confirmed a significantly higher kidney fluorescence signal in SeZM NP‐treated mice (Figure S7B, Supporting Information). Furthermore, ICP‐MS measurements of major organs at 12 h post‐injection showed elevated selenium content in the kidneys of SeZM NPs‐treated mice compared to SeZ NPs‐treated ones (Figure S7C,D, Supporting Information), highlighting their enhanced renal tropism of SeZM NPs, which may be associated with the renal enrichment of M2b macrophages in LN.

### 2.10 SeZM NPs Alleviate Renal Inflammation and Protect Lupus Mice from Renal Damages

**Figure 6:**
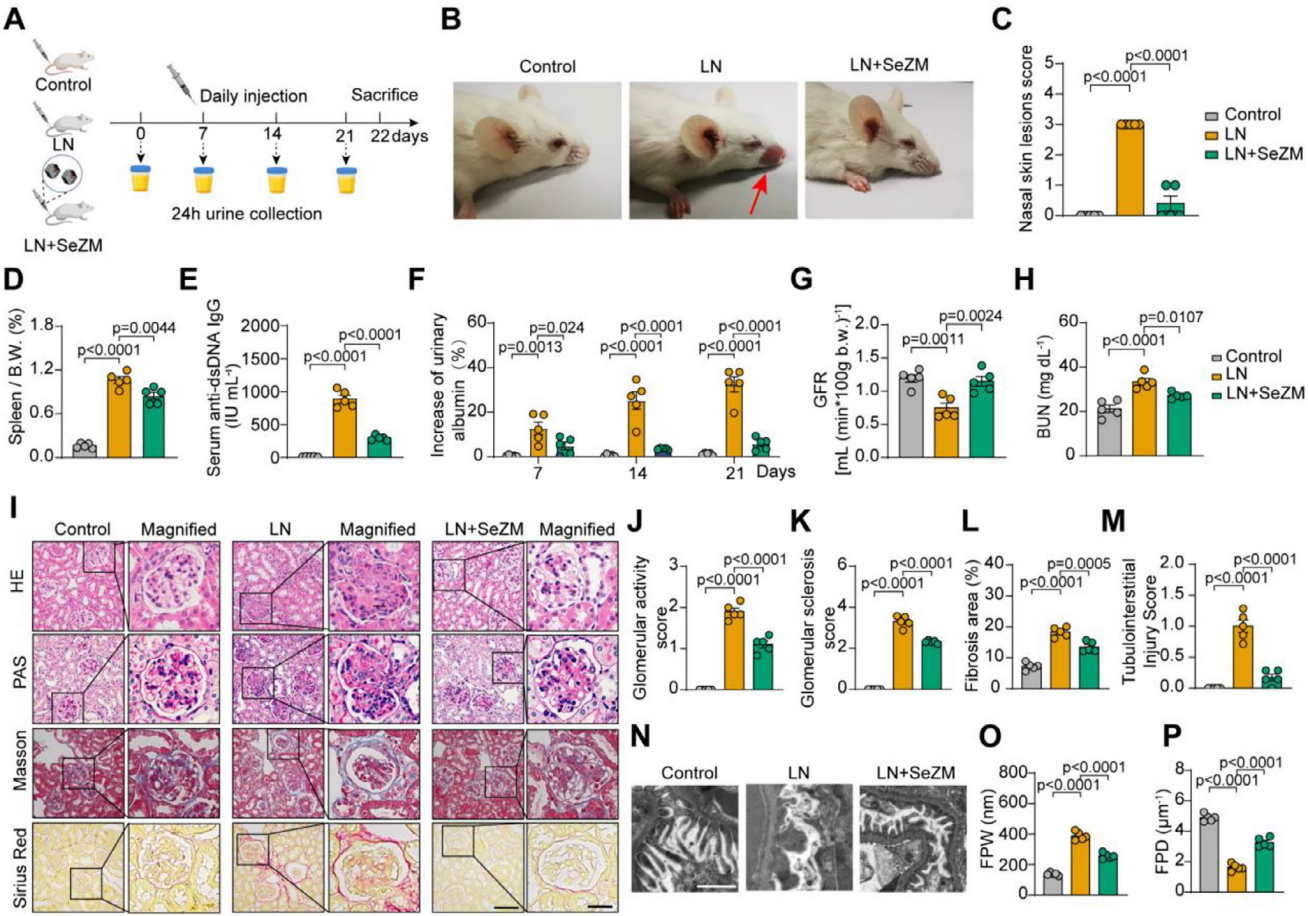
SeZM NPs attenuate renal injury in MRL/lpr lupus mice. A) Experimental scheme. Control: MRL/MpJ mice injected with saline; LN: MRL/lpr mice injected with saline; LN+SeZM: MRL/lpr mice injected with SeZM NPs (1 mg kg^−1^). Treatments were administered intravenously once daily for 21 days. B,C) Representative images of mice on day 21 (B) and nasal skin lesion scores (C). The red arrow indicates cutaneous damage. D) Spleen‐to‐body weight ratios from three groups. E) Serum anti‐dsDNA IgG levels on day 21 from three groups. F) The increase in 24 h urinary albumin levels from three groups at days 7, 14, and 21 compared to day 0. G) Glomerular filtration rate (GFR) measured by transdermal detection of FITC‐sinistrin clearance on day 21. H) Serum blood urea nitrogen (BUN) levels from three groups. I) Representative kidney histology (H&E, PAS, Masson, and Sirius Red staining) from three groups. Scale bar, 100 µm; scale bar of magnified area, 40 µm. J–M) Quantification results of glomerular activity (j), glomerular sclerosis (K), fibrotic area (L), and tubulointerstitial injury (M). N) Transmission electron microscopy of podocyte foot processes. Scale bar, 1 µm. O,P) Quantification of foot process width (FPW; O) and foot process density (FPD; P) based on (N).

Encouraged by the M2b macrophage‐targeting capability and kidney retention of SeZM NPs, we then evaluated their therapeutic efficacy in vivo using the MRL/lpr mouse model of LN. MRL/lpr mice were intravenously administered SeZM NPs at a concentration of 1 mg kg^−1^ daily for 21 days (LN+SeZM group), with saline‐treated MRL/MpJ and MRL/lpr mice serving as controls and LN groups, respectively (**Figure** **6**A). Importantly, SeZM NPs did not alter body weight, indicating favorable biocompatibility in vivo (Figure S8, Supporting Information), and significantly attenuated nasal skin lesions, an external hallmark of autoimmune severity in lupus mice (Figure 6B,C). Moreover, SeZM NPs treated LN mice exhibited reduced spleen‐to‐body weight ratios and serum anti‐dsDNA IgG levels (Figure 6D,E), two established markers of systemic lupus activity. As expected, SeZM NPs significantly mitigated the progressive rise in 24 h urinary albumin (Figure 6F), improved glomerular filtration rate (GFR), and reduced blood urea nitrogen (BUN) levels in lupus mice (Figure 6G,H), suggesting the strong protective effect of SeZM NPs on renal functions.

Histopathological analyses demonstrated that SeZM NPs treatment alleviated glomerular hypercellularity, sclerosis, interstitial fibrosis, and tubular injury as observed by H&E, PAS, Masson, and Sirius red staining (Figure 6I). Quantitative analysis of histology confirmed significant improvements in glomerular activity, glomerular sclerosis, fibrotic area, and tubulointerstitial injury (Figure 6J–M). Moreover, TEM revealed that SeZM NPs effectively reversed podocyte foot process effacement (FPE), as reflected by reduced foot process width (FPW) and increased foot process density (FPD) (Figure 6N–P), indicating structural protection at the ultrastructural level of SeZM NPs. Together, these results demonstrate that SeZM NPs exert potent renoprotective effects in LN mice, ameliorating both systemic autoimmunity and local kidney pathology.

**Figure 7:**
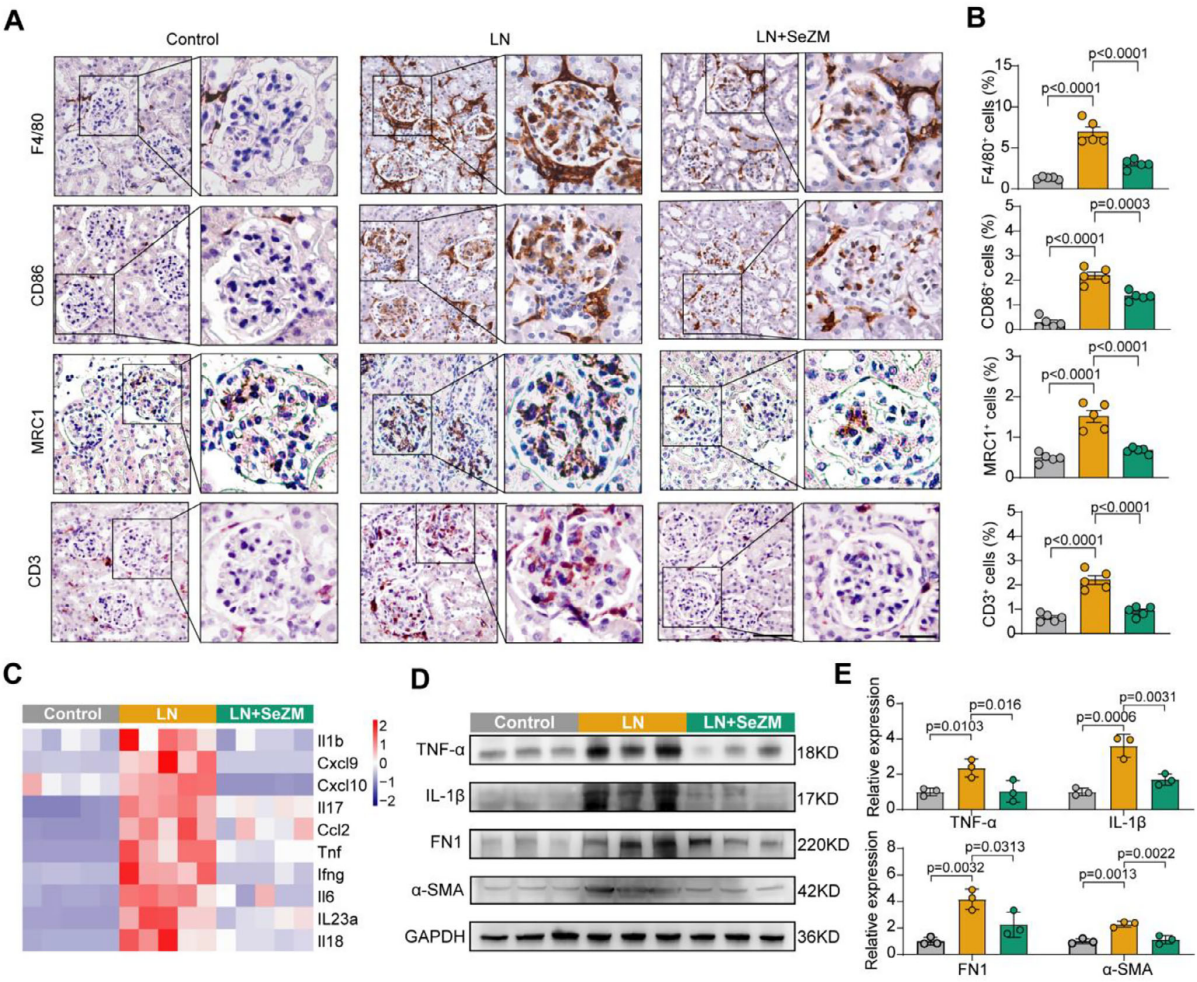
SeZM NPs alleviate renal inflammation and fibrosis in lupus mice. A‐B) Representative immunohistochemical images A) and quantification results B) of F4/80⁺, CD86⁺, MRC1⁺ (CD206), and CD3⁺ cells in the kidneys from three groups. Control group: MRL/MpJ mice treated with saline; LN group: MRL/lpr mice treated with saline; LN + SeZM group: MRL/lpr mice treated with SeZM NPs (1 mg kg^−1^). Scale bar: 100 µm; scale bar of magnified area: 40 µm. C) RT‐PCR analysis of the mRNA expression levels of pro‐inflammatory cytokines and chemokines in kidney tissues from the three groups. D,E) Representative western blotting results D) and quantification results E) of TNF‐α, IL‐1β, fibronectin 1 (FN1), and α‐SMA protein expression in kidney tissues from three groups.

To investigate whether the renoprotective effect of SeZM NPs was due to the immune regulation of M2b macrophages in LN mice, we carried out immunohistochemical staining for M2b macrophages and T cells. Immunohistochemical staining revealed marked reductions in F4/80⁺ macrophages and CD86^+^MRC1+ M2b macrophages in SeZM NPs‐treated LN mice compared to PBS‐treated LN mice. Furthermore, the number of CD3^+^ T cells, which are mainly recruited by activated macrophages, was also significantly reduced in LN mice treated with SeZM NPs (**Figure** **7**A,B). The expression levels of major LN‐associated pro‐inflammatory cytokines and chemokines were significantly increased in the LN group compared to the control group. Treatment with SeZM NPs effectively suppressed the expression of these genes in LN mice (Figure 7C). At the protein level, SeZM treatment diminished expression of TNF‐α, IL‐1β, and fibrotic markers fibronectin (FN1) and α‐smooth muscle actin (α‐SMA) (Figure 7D,E). These findings indicate that SeZM NPs mitigate renal inflammation and fibrosis by targeting M2b macrophages, thereby interrupting the downstream proinflammatory cascade in lupus nephritis.

## 3 Conclusion

Lupus nephritis is a severe complication of SLE.^[^ ^32^ ^]^ It is characterized by the accumulation of immune complexes and the activation of immune cells, especially macrophages, in the kidneys.^[^ ^33^, ^34^ ^]^ Previous studies and our scRNA‐seq analysis indicate that the progression of LN is closely associated with a specific population of macrophages, the pro‐inflammatory M2b macrophages. However, no therapeutic strategies have been developed to specifically target this pathogenic population. Selenium is a micronutrient that is essential for human health and plays a specific role in regulating the immune system.^[^ ^35^, ^36^, ^37^ ^]^ Through the interaction with various selenoprotein families, selenium has been shown to regulate the immune cell functions.^[^ ^38^, ^39^, ^40^ ^]^ We therefore developed a selenium@ZIF‐8@Mannoside nanoparticles (SeZM NPs) that could selectively target M2b macrophages via the mannose receptor MRC1 (CD206) and regulate their polarization and activation. The synthesized SeZM NPs exhibited favorable biocompatibility, low cytotoxicity, and high targeting efficiency toward M2b macrophages both in vitro and in vivo.

It is noteworthy that SeZM NPs were effective in reducing the expression levels of pro‐inflammatory cytokines and chemokines in M2b macrophages. In addition, these nanoparticles exhibited a strong antioxidant capacity, efficiently reducing the production of ROS and preventing a decrease in the mitochondrial membrane potential in M2b macrophages. Transcriptomic analysis revealed that SeZM NPs suppressed the polarization and activation of M2b macrophages by regulating the expression of selenoproteins, including GPX1, which is an important protein for intracellular ROS clearance. In animals, SeZM NPs exhibited an increased enrichment level in the kidneys of lupus mice, and significantly reduced the levels of M2b macrophage‐associated pro‐inflammatory cytokines and chemokines, which in turn effectively protect kidney structure and restore kidney function. Taken together, our results demonstrated the therapeutic potential of selenium‐based nanoparticles for the treatment of lupus nephritis as well as the M2b macrophage‐related autoimmunity.

## 4 Experimental Section

### Materials, Cell Lines and Animals

PEG‐400 (Cat#P103737), hydrogen peroxide (H2O2, Cat#H433860), and Zn(NO3)2·6H2O (Cat#Z111703) were purchased from Aladdin Company (Shanghai, China). Se powder (Cat#S817647), 2‐Methylimidazole (Cat#M813135), Indocyanine Green (ICG, Cat# I953656), D‐Mannose (Cat#D813082), and Polyvinyl pyrrolidone (Cat# P816205) were purchased from Macklin Inc. (Shanghai, China). DMPO (Cat# D0) and CCK‐8 cell viability kits (Cat#CK04) were purchased from Dojindo Company (Kumamoto, Japan). Proteinase K (Cat#03654672103) was purchased from Roche Inc (Basall, Switzerland). Dulbecco's modified Eagle's medium (DMEM) (Cat#C11995500BT), RPMI‐1640 medium (Cat#11875‐093), and penicillin‐streptomycin (PS) (Cat#15140122) were purchased from Gibco (New York, USA). Mouse M‐CSF protein (Cat#416 mL) was purchased from R&D Systems (Minneapolis, USA). Fetal bovine serum (FBS) (Cat#S‐FBS‐SA‐015) was purchased from Serena (Brandenburg, Germany). LPS (Cat#tlrl‐pb5lps) was purchased by InvivoGen (New York, USA). APC‐anti‐mouse CD11b (Car#553312), BV421‐anti‐mouse CD86 (Cat#564198), PerCP/Cyanine5.5‐anti‐mouse CD206 (Cat#141716), and PerCP‐Cyanine5.5‐anti‐mouse CD45 (Cat#550994) were purchased by BD Biosciences (New Jersey, USA). Calcein/PI cell viability/cytotoxicity assay kit (Cat#C2015M), Hoechst 33342 assay kits (Cat#C1028), and BeyoBlue staining solution (Cat#P0017F) were purchased from Beyotime (Shanghai, China). Annexin V apoptosis detection kits (Cat#88‐8006‐74) were purchased by eBioscience (San Diego, USA). Anti‐TNF alpha (Cat#ab1793), Anti‐F4/80 (Cat#ab300421), Anti‐Mannose Receptor (Cat#ab300621), and Anti‐IL‐1 beta (Cat#ab254360) antibodies were purchased from Abcom (New York, USA). Anti‐IL‐6 (Cat#A24522), Anti‐JAK1 (Car#11953), Anti‐phospho‐JAK1 (Cat#AP1406), Anti‐JAK2 (Cat#19629), Anti‐STAT1 (Cat#A19563), and Anti‐phospho‐STAT1 (Cat#AP0054) antibodies were purchased from Abclonal (Wuhan, China). Anti‐phospho‐JAK2 (Cat#3771S), Anti‐CD86 (Cat#19589S), HRP conjugated goat anti‐rabbit IgG (Cat#7074S), and HRP conjugated horse anti‐mouse IgG (Cat#7076S) antibodies were purchased from Cell Signaling Technology (Danforth, USA). HRP‐conjugated GAPDH (Cat#60004) antibodies were purchased from Proteintech (Wuhan, China). Anti‐CD3 (Cat#A0452) antibodies were purchased from Agilent (Beijing, China). Anti‐Fibronectin (Cat#BA1772) was purchased by Bioss (Massachusetts, USA). ROS Assay Kit (Cat#CA1410) was purchased by Solarbio (Beijing, China). JC‐1 staining kits (Cat#22200) were purchased by AAT Bioquest (Pleasanton, USA).

Raw264.7 cells (RRID: CVCL_0493, Cat# CL‐0190), THP‐1 cells (RRID: CVCL_0006, Cat# CL‐0233), and Jurkat cells (RRID: CVCL_0367, Cat# CL‐0129) were purchased from Procell (Wuhan, China). All cell lines were authenticated by the supplier and tested negative for mycoplasma contamination prior to use.

Female C57BL/6 mice between 6 and 8 weeks were purchased from Guangdong Medical Laboratory Animal Center (China). Female MRL/MpJ and MRL/lpr mice aged 13 weeks were sourced from Huachuang SinoPharmaTech Co., Ltd (China). All animal experiments were performed in accordance with the Guide for the Care and Use of Laboratory Animals and approved by the Institutional Animal Care and Use Committee of Sun Yat‐sen Memorial Hospital (Animal ethics ID: AP20240106). Formalin‐fixed, paraffin‐embedded renal tissue sections were obtained from patients undergoing nephrectomy for renal cell carcinoma (adjacent normal tissue) and from lupus nephritis patients via kidney biopsy, with approval from the Institutional Ethics Committee of Jieyang People's Hospital (Human ethics ID: 2025052).

### Synthesis and Characterization of SeZM NPs

### Synthesis and Characterization of SeZM NPs—Synthesis of SeNPs

Selenium powder (20 mg) was dissolved in a PEG‐400 solution (10 mL) and ultrasonically dispersion of selenium powder in a 50 mL beaker. Then, the PEG‐400 and selenium powder mixture solution was moved to a magnetic stirring heater. The magnetic stirring heater heated the mixed solution and monitored the mixed solution with a thermometer to keep the temperature of the mixed solution at 220 °C. After 1 h, a brown mixed solution was obtained and set at room temperature. Finally, the SeNPs were obtained.

### Synthesis and Characterization of SeZM NPs—Synthesis of DSPE‐PEG‐Man

To prepare the PEG solution, PEG‐COOH (Mn: 5000 Da) was fully dissolved in 8 mL of water under stirring. Separately, NHS (25 mg), EDC (25 mg), and D‐mannose (50 mg) were dissolved in 2 mL of water. The mannose‐containing solution was then added dropwise to the DSPE‐PEG solution, and the mixture was stirred overnight at 25 °C. The resulting solution was then subjected to dialysis against water for 24 h, with the purpose of removing any unreacted reagents. This process resulted in the production of DSPE‐PEG‐Man.

### Synthesis and Characterization of SeZM NPs—Synthesis of SeZ NPs

To synthesize SeZ NPs, SeNPs (3 mL) and PVP (200 mg) were added to 20 mL of a 2‐methylimidazole solution (42 mg dissolved in methanol) under stirring until completely dissolved. Then, 20 mL of zinc nitrate hexahydrate solution (150 mg in methanol) was added, and the mixture was left to stand at 25 °C for 6 h.^[^ ^41^ ^]^ The precipitate was harvested by centrifugation (12 000 rpm, 10 min) and subsequently washed three times with methanol. The purified SeZ NPs were redispersed in 8 mL of deionized water.

### Synthesis and Characterization of SeZM NPs—Synthesis of SeZM NPs

To prepare SeZM NPs, 2 mL of DSPE‐PEG‐Man solution was added to the SeZ NPs dispersion and stirred at 25 °C for 12 h. The mixture was then centrifuged at 12 000 rpm for 10 min, and the precipitate was washed three times with deionized water to obtain the final SeZM NPs.

### Synthesis and Characterization of SeZM NPs—Characterization of Nanoparticles

The topography and structure of nanoparticles were characterized using a TEM (H‐7650, Hitachi High‐Tech, Japan) operated at 100 kV and an HR‐TEM (JEM‐2010HR, JEOL Ltd., Japan). Energy‐dispersive X‐ray was performed in HR‐TEM to analyze the elemental mapping of the SeZM NPs. UV–vis–NIR absorption spectra were recorded using a UH‐4150 spectrophotometer (Hitachi High‐Tech, Japan). Fluorescence emission spectra were measured with a LUMINA fluorescence spectrometer (Thermo Fisher Scientific, USA). X‐ray photoelectron spectroscopy (XPS) was conducted using a K‐Alpha+ spectrometer (Thermo Fisher Scientific, USA). These techniques were used to characterize the optical properties and surface chemical composition of SeNPs, ZIF‐8, SeZ NPs, and SeZM NPs.

### Assessment of ROS Scavenging Activity

The total ROS scavenging ability of different selenium species was detected using the ABTS method with a total antioxidant capacity assay kit (Beyotime Biotechnology, China). Hydroxyl radicals (·OH) were generated via the Fenton reaction using Fe^2^⁺ (5 mM) and H_2_O_2_ (5 mM). The superoxide radical (·O_2_^−^) was obtained by the reaction system of KO_2_ (0.7 mg mL^−1^) and 18‐Crown‐6 (6 mg mL^−1^). Then, the hydroxyl radical and superoxide radical scavenging abilities of SeZM NPs were detected by electronic spin resonance (ESR5000, Magnettech, Germany).

### Selenium Metabolite Assay

BMDMs (8 × 10^6^) treated with 10 µg mL^−1^ SeZM NPs for 0, 2, 4, 8, 12, and 24 h and then were collected and digested with 4 mL digestion solution containing trypsin (4 mg mL^−1^) and Proteinase K (4 mg mL^−1^). Cell lysates were then subjected to ultrasonication on an ice bath, followed by incubation at 37 °C for 24 h. Subsequent centrifugation was performed at 12 000 × g for 15 min. The resulting supernatant was filtered through a 220 nm membrane and analyzed using HPLC–ICP–MS with ammonium citrate buffer (10 mmol L^−1^) as the mobile phase, running at a flow rate of 1 mL min^−1^. For the quantification of the metabolites of SeZM NPs in the BMDMs, the following standards were used: selenite (SeO_3_2^−^), selenate (SeO_4_2^−^), selenomethionine (SeMet), selenocystine (SeCys_2_), and methylselenocystine (MetSeCys) were used as standards.

The resulting supernatant was filtered through a 220 nm membrane and analyzed using HPLC–ICP–MS with ammonium citrate buffer (10 mmol L^−1^) as the mobile phase, running at a flow rate of 1 mL min^−1^.

### BMDMs Isolation and Cell Culture

Fresh BMDMs were obtained from C57BL/6 mice using well‐established protocols.^[^ ^41^ ^]^ In brief, bone marrow cells were isolated from euthanized mice via PBS flushing, and erythrocytes were subsequently eliminated using lysis buffer. The remaining mononuclear cells were cultured in DMEM supplemented with M‐CSF (20 ng mL^−1^), 10% FBS, and 1% penicillin‐streptomycin (PS) for 7 days to generate BMDMs. RAW 264.7 cells were cultured in DMEM, while THP‐1 and Jurkat cells were maintained in RPMI‐1640. All culture media were supplemented with 10% FBS and 1% PS, and cells were maintained at 37 °C in a humidified incubator with 5% CO_2_.

### M2b Macrophage Polarization

M2b macrophages were induced using a standard protocol by treating BMDMs with lipopolysaccharide (LPS, 2 ng mL^−1^) and immune complexes (ICs, 50 µg mL^−1^) for 24 h. The ICs were generated by pre‐incubating ovalbumin (OVA, 100 µg mL^−1^) with anti‐OVA IgG (100 µg mL^−1^) for 1 h to allow immune complex formation.

### Flow Cytometry Assay

Cells were pelleted by centrifugation, resuspended, and labeled with specific antibodies or fluorochrome‐conjugated probes for flow cytometric analysis. These included Annexin V, 7‐AAD, CD11b‐APC, CD86‐BV421, CD206‐PerCP/Cyanine5.5, CD45‐APC‐Cy7, CD3‐APC, CD19‐FITC, NK1.1‐FITC, and 2′,7′‐dichlorodihydrofluorescein diacetate (DCFH‐DA; Cat# CA1410, Solarbio, China). Samples were incubated in the dark for appropriate durations following the manufacturer's protocols. The fluorescence‐positive populations were analyzed using a BD FACSVerse flow cytometer (BD Biosciences, USA).

### Intracellular ROS Production Assay

DCFH‐DA was employed as a fluorescent probe to assess intracellular ROS levels.^[^ ^42^ ^]^ Briefly, M0 and M2b BMDMs (3 × 10^5^ per well) were plated on µ‐slide bioinerts (Cat#80420, iBiDi, Germany) and cultured overnight. Subsequently, ZM NPs or SeZM NPs (10 µg mL^−1^) were then added to M2b macrophages for 4 h. Subsequently, the culture media were extracted, and the treated BMDMs were carefully rinsed with PBS. The BMDMs were then placed in PBS containing DCFH‐DA (10 µM) and Hoechst 33342 (5 µg mL^−1^) for 30 min in the absence of light. Finally, macrophages were imaged by using a fluorescent microscope, and the mean fluorescence intensity of ROS was quantified by ImageJ.

### JC‐1 Staining Assay

M0 or M2b BMDMs (3 × 10⁵ cells per well, n = 4) were seeded into µ‐slide bioinert chambers and incubated overnight. ZM NPs or SeZM NPs (10 µg mL^−1^) were then added to the culture medium of M2b macrophages for 12 h. Cells were incubated in the dark for 30 min with JC‐1 (20 µg mL^−1^; 5,5′,6,6′‐tetrachloro‐1,1′,3,3′‐tetraethyl‐benzimidazolocarbocyanine iodide) and Hoechst 33342 (5 µg mL^−1^) following PBS washing. Fluorescence microscopy was used to acquire images, and the fluorescence intensity was quantified using ImageJ.

### Seahorse Assay

Raw264.7 cells (8 × 10⁴ per well) were seeded into XF96 cell culture microplates (Cat#102601, Seahorse Bioscience, USA) and polarized into M2b macrophages using standard protocols.^[^ ^43^ ^]^ After polarization, cells were treated with SeZM NPs (10 µg mL^−1^) for 6 h. After replacing the culture medium with Seahorse assay buffer, cells were incubated at 37 °C for 90 min in a CO_2_‐free environment. Mitochondrial respiration was evaluated using the XF Cell Mito Stress Test Kit (Agilent Technologies, USA), following sequential addition of oligomycin (1.0 µM), Carbonyl cyanide 4‐(trifluoromethoxy)phenylhydrazone (FCCP, 1.5 µM), and rotenone/antimycin A (0.5 µM). The oxygen consumption rate (OCR) was recorded using the Seahorse XF96 Analyzer.

### RNA‐Sequencing

To investigate transcriptomic changes, macrophages were divided into three experimental groups: M0 macrophages treated with PBS (Control), M2b macrophages treated with PBS (M2b), and M2b macrophages treated with SeZM NPs (10 µg mL^−1^) (M2b+SeZM). TRIzol reagent was used to extract RNA from macrophages, followed by mRNA enrichment via poly‐A selection. First‐ and second‐strand cDNA were synthesized using oligo (dT) primers and standard enzymes. Following end repair and size selection (≈370–420 bp), libraries were constructed, amplified, and quality‐checked. Sequencing was performed on the Illumina NovaSeq 6000 platform.

### Pathway‐Gpx1 Correlation Analysis

A pathway‐based correlation analysis using normalized RNA‐seq data was performed to investigate the functional relevance of Gpx1. Gene members of the JAK‐STAT, PI3K‐Akt, and MAPK signaling pathways were obtained from the KEGG canonical pathway collection. For each sample, pathway activity was estimated by calculating the arithmetic mean of log2‐transformed expression values (log2[TPM + 1]) of all genes within each pathway. Pearson correlation coefficients between Gpx1 expression and pathway activity scores were computed across samples using the Hmisc package in R, followed by Benjamini–Hochberg correction for multiple testing, as described previously.^[^ ^44^ ^]^

### Nasal Skin Lesions Assessment

Nasal skin lesions were assessed in MRL/lpr mice using a semi‐quantitative scoring system adapted from previously published protocols with minor modifications.^[^ ^45^ ^]^ Lesions were evaluated based on the severity of erythema, alopecia, crusting, and ulceration in the nasal area, and graded on a scale from 0 to 4. A score of 0 indicated intact skin with no visible lesions, redness, or hair loss. Mild erythema or limited alopecia confined to the nasal tip corresponded to a score of 1. A score of 2 represented more apparent erythema with moderate alopecia extending across the nasal bridge, with possible roughening of the skin. Pronounced erythema and alopecia accompanied by crusting or scaling but without ulceration were graded as 3. The most severe lesions, characterized by extensive erythema, substantial alopecia, visible crusting, and ulceration or exudation, received a score of 4.

### 24 h Urinary Albumin Assay

Mice were placed in metabolic cages supplied with only water for 24 h. Urine samples were centrifuged to remove debris and then quantified. A volume of urine (500 µL) was taken and then mixed with methanol (500 µL) and chloroform (120 µL), and centrifuged at 13 000 rpm for 10 min to perform protein purification. Additional methanol was added to improve protein precipitation. The urinary total proteins were denatured and separated by electrophoresis on SDS‐PAGE gels with albumin standards (Cat#23209, Thermofisher). After staining with Coomassie brilliant blue (Cat#P0017F, Beyotime, China), the amount of urinary albumin of each mouse was calculated using the albumin standard curve.

### GFR Measurement

GFR was measured using a transcutaneous fluorescent tracer method described in previous research.^[^ ^46^, ^47^ ^]^ Briefly, mice were anesthetized with isoflurane and mounted on a compact fluorescence detector (MediBeacon GmbH, Germany). The detector was fixed to the shaved and hairless region of the mouse's back using double‐sided adhesive tape. Prior to intravenous injection of 70 mg kg^−1^ FITC‐sinistrin (MediBeacon GmbH, Germany), the background signal from the skin was collected for 5 min. The mice were allowed to recover and were individually housed for 1 h. GFR values were calculated by fitting the decay of FITC‐sinistrin fluorescence intensity to a single‐compartment model, taking into account plasma half‐life and body weight. Data analysis was performed using the MB Lab/MB Studio software (MediBeacon GmbH, Germany).

### Renal Pathology Scoring

Glomerular injury was evaluated using two indices: an activity score and a sclerosis score.^[^ ^48^ ^]^ The glomerular activity score was determined based on the proportion of the glomerular tuft exhibiting acute inflammatory features, including hypercellularity, leukocyte infiltration, fibrinoid necrosis, and crescent formation, and graded as follows: 0 = none; 1 =< 25%; 2 = 25%–50%; 3 = 50%–75%; 4 = >75%. The glomerular sclerosis score was based on the percentage of the tuft replaced by sclerotic lesions, including mesangial matrix expansion or obliterated capillary loops with dense PAS‐positive material, and scored on the same 0–4 scale.

Tubulointerstitial injury was assessed on PAS‐stained sections according to the extent of tubular dilatation, epithelial injury, basement membrane irregularity or thickening, intraluminal cast formation, and interstitial inflammatory infiltration.^[^ ^49^ ^]^ A semi‐quantitative scale was used: 0 = none; 1 =< 25%; 2 = 25%–50%; 3 = >50% of the cortical field affected. Scores from 10 random ×200 fields were averaged per kidney.

Renal fibrosis was evaluated on Masson's trichrome and Sirius red–stained sections. The fibrotic area was quantified as the percentage of positively stained (blue or red) area in the renal cortex using ImageJ, averaged from multiple random ×200 fields per sample.

### Western Blotting Assay

Proteins were extracted using RIPA lysis buffer, and concentrations were assessed using a Bradford assay. Equal amounts of protein were separated by SDS–PAGE and transferred to PVDF membranes. After blocking, membranes were incubated with primary and HRP‐conjugated secondary antibodies. Chemiluminescent signals were developed and imaged, and band intensities were quantified using ImageJ.

### RT‐PCR

Total RNA was obtained using an RNA Extraction Kit (Cat#RN001, ESscience, China) according to the manufacturer's instructions. Subsequently, cDNA was prepared using a PrimeScript RT Master Mix (Cat#RR06A, TAKARA, Japan). The RT‐PCR amplification was performed on an Azure Cielo 3 system using the SYBR Green fluorescence detection method. The obtained data were analyzed using CqMANs.

### Statistical Analysis

The data were reported as the mean ± standard error of the mean (SEM) for statistical analysis. Statistical comparisons between two groups were performed using an unpaired two‐tailed Student's *t*‐test. For comparisons among three or more groups, one‐way ANOVA followed by Tukey's post hoc test was used. *p*‐values less than 0.05 were considered statistically significant. All analyses were performed using GraphPad Prism.

## Conflict of Interest

The authors declare no conflict of interest.

## Author Contributions

H. L., G. H., H. L., and H. L. contributed equally to this work. Q.L., Y.Z., T.C., H.L., G.H., and H.L. designed the experiments. Q.L., Y.Z., T.C., and G.H. funded this study. T.C. and G.H. designed and synthesized the selenium nanoparticles. H.L., H.L., and H.L. conducted the experiments in vitro. H.L., D.Z., K.M., H.P., Y.D., and S.L. conducted and analyzed the single‐cell sequencing and RNA‐seq experiment. H.L., G.H., H.L., H.L., Y.D., D.Z., and K.M. conducted the experiments in vivo. K.W. and W.C. provided human pathological sections and clinical data. Y.Z., H.M., and W.C. provided technical support and participated in data analysis. H.L., G.H., and Y.Z. contributed to the experimental discussion. H.L., G.H., H.L., and H.L. generated the figures, and all the authors participated in the writing. All authors agree to publish the manuscript.

## Supporting information



Supporting Information

Supporting Information

## Data Availability

The data that support the findings of this study are available from the corresponding author upon reasonable request.
