# Supplementary material for: Reprogramming M2b Macrophages via GPX1 Activation by Selenium Nanoparticles Attenuates Lupus Nephritis
Source: Adv Sci (Weinh). 2025 Dec 17;13(12):e19981. doi: 10.1002/advs.202519981 (PMC12948211; doi:10.1002/advs.202519981)
Supplement: Supplementary file 1 — Supporting Information [file ADVS-13-e19981-s001.docx]

**Supporting Information**

# Reprogramming M2b Macrophages via GPX1 Activation by Selenium Nanoparticles Attenuates Lupus Nephritis

*Haoran Lv^1,2,3#^, Guanning Huang^4#^, Hongyu Li^1,2,3#^, Hanzhi Liang^1,2,3#^, Huajing Peng^1,2^, Kefei Wu^3^, Wenfang Chen^5^, Dandan Zhang^1,2^, Kexin Ma^1,2^, Yufei Du^6^, Siweier Luo^6^, Yi Zhou^1,2^, Haiping Mao^1,2^, Wei Chen^1,2^, Tianfeng Chen^4*^, Yiming Zhou^6*^, Qinghua Liu^1,2,3*^*

1. Department of Nephrology, The First Affiliated Hospital, Sun Yat-sen University, Guangzhou, China
2. NHC Key Laboratory of Clinical Nephrology (Sun Yat-sen University) and Guangdong Provincial Key Laboratory of Nephrology, Guangzhou, China
3. Department of Nephrology, Jieyang People's Hospital, Jieyang, China
4. State Key Laboratory of Bioactive Molecules and Druggability Assessment, MOE Key Laboratory of Tumor Molecular Biology, Department of Chemistry, Jinan University, Guangzhou, China
5. Department of Pathology, The First Affiliated Hospital, Sun Yat‑sen University, Guangzhou, China
6. Basic and Translational Medical Research Center, Sun Yat-sen Memorial Hospital, Sun Yat-sen University, Guangzhou, China

^#^These authors contributed equally to this work. ^*^Corresponding authors. ^$^Current address: Department of Nephrology, The First Affiliated Hospital, Sun Yat-sen University, Guangzhou, China

Corresponding E-mail: liuqhua6@mail.sysu.edu.cn (Qinghua Liu), zhouym35@mail.sysu.edu.cn (Yiming Zhou), and tchentf@jnu.edu.cn (Tianfeng Chen).

**Supplementary Methods**

**Biocompatibility Assays**

After removing the plasma and leukocytes, RBCs were diluted to 10% concentration with PBS. As a hemolytic control, double distilled water was applied to the RBCs until hemolysis occurred. Different amounts of SeZM NPs (0, 10, 20, 40, and 80 μg mL^-1^) were added to the 10% RBC solution and incubated at 37°C for 2, 4, 8, 12, and 24 h, respectively. The cells were photographed using an inverted light microscope (Nikon, TI-S). The degree of hemolysis was investigated through measurement of the absorbance of the RBCs supernatants at 540 nm, with a hemolysis ratio subsequently determined using the following formula:

Hemolysis ratio= [(Experimental group - Negative control group) x (Positive control group - Negative control group)-1]x100%

Negative control group: RBCs treated with PBS;

Positive control group: RBCs treated with ddH2O.

**Cell Viability Assay**

CCK-8 assays were performed following the instructions provided by the manufacturer. In brief, BMDMs (2.5x10^4^ per well) were placed in 96-well plates and subjected to varying concentrations of SeZM NPs (0, 5, 10, 15, 20, and 30 μg mL^-1^) for either 12 or 24 h. CCK-8 solution (100 μL) was then added and incubated for 2 h at 37°C in the absence of light. Absorbance was measured at a specific wavelength of 450 nm using a multimode microplate reader (Bioteck Synergy H1, Agilent).

BMDMs (8x10^5^ per well) were cultured on six-well plates and incubated overnight. Different doses of SeZM NPs (0, 10, 20, and 30 μg mL^-1^) were then added to the culture medium and incubated for 24 h. After removal of the culture medium and washing with PBS, calcein (2 μM) and PI (4.5 μM) was added for 30 min to label live and dead macrophages, respectively. The labeled cells were quantified by a fluorescence microscope (ECLIPSE Ts2R-FL, Nikon).

***In Vitro* Chemotactic Assay**

THP-1 cells (3x10^5^ per well) were seeded in a lower transwell chamber (Cat#4395, Corning, USA) treated with 150 ng mL^-1^ phorbol-12-myristate-13-acetate (PMA, Cat#P8139, Sigma, USA) for 24 h to convert them into M0 macrophages (n=5). Subsequently, these cells were incubated with LPS (100 ng mL^-1^) and immune complexes (30 μl mL^-1^) together with ZM NPs or SeZM NPs (10 μg mL^-1^) for 24 h. The immune complexes were prepared by a conventional method.^[48]^ Jurkat cells (5x10^5^ per well) were then introduced to an upper chamber of the transwell plate. The Jurkat cells migrated to the lower compartment were determined after 24 hours.

**Targeting Assays of nanoparticles *in vitro***

BMDMs were polarized into five canonical subtypes prior to nanoparticle uptake assessment. M0 macrophages were cultured without stimulation. M1 macrophages were induced by 2 ng mL⁻¹ LPS. M2a macrophages were generated using interleukin-4 (IL-4, 20 ng mL⁻¹) and interleukin-13 (IL-13, 20 ng mL⁻¹), while M2c macrophages were induced with interleukin-10 (IL-10, 20 ng mL⁻¹). All stimulations were maintained for 24 h before nanoparticle administration.

To evaluate nanoparticle uptake, Rhodamine B-labeled SeZ NPs and mannose-modified SeZM NPs (10 μg mL⁻¹) were incubated with each macrophage subtype for 3 h at 37 °C. After incubation, cells were stained with Hoechst 33258 (5 μg mL⁻¹; Beyotime, China) for 30 min to visualize nuclei. Fluorescence images were acquired using a confocal microscope to assess the percentage of PE⁺ macrophages. Quantification was performed using ImageJ.

**Targeting Assays of nanoparticles *in vivo***

To evaluate the *in vivo* biodistribution and immune cell targeting efficiency of nanoparticles, MRL/lpr mice were randomly assigned to receive intravenous injections of PBS (control), Rhodamine B-labeled SeZ NPs (1 mg kg⁻¹), or SeZM NPs (1 mg kg⁻¹) via the tail vein. At 4 h post-administration, spleens were harvested, and single-cell suspensions of splenocytes were prepared by mechanical dissociation and red blood cell lysis.

Flow cytometry was conducted to determine the uptake of Rhodamine B-labeled nanoparticles in various immune cell populations. Cells were stained with fluorophore-conjugated antibodies against CD3 (T cells), CD19 (B cells), NK1.1 (NK cells), and CD11b (macrophages), all in combination with CD45 to ensure gating on leukocytes. For macrophage subset analysis, cells were further stained for CD86 and CD206 to identify M2b macrophages (CD86⁺CD206⁺). Rhodamine B positive (PE⁺) cells were quantified using a BD Fortessa flow cytometer and analyzed with FlowJo software.

***In vivo* Fluorescence Imaging Assay in MRL/lpr mice**

The ICG-labeled SeZM NPs were manufactured using a slightly modified method compared to the process utilized for synthesizing the SeZM NPs. During the synthesis of SeZM NPs, ICG (0.5 mg mL^-1^) was moved to the 2-Methylimidazole solution to encapsulate ICG during the metal organic framework synthesis process. The fluorescence production was centrifuged at 12000 rpm for 10 min, washed with methanol, and finally ICG-labeled SeZM NPs and SeZ NPs were obtained. The ICG-labeled SeZM NPs (0.4 mg kg^-1^) were intravenously injected into the MRL/lpr mice. Fluorescence was monitored with an imaging system (NightOWL II LB 983, Berthold) at 0, 0.25, 0.5, 1, 2, 4, 8, and 12 h. Fluorescence intensity was also quantified using ImageJ. After 12 h of in vivo fluorescence imaging, mice were euthanized and major organs, including heart, liver, spleen, lungs, and kidneys were collected and digested with mixed acid (Vnitric acid: Vperchloric acid = 3:1). The selenium concentration in each organ was determined using an atomic fluorescence spectrometer (iCAP RQ, ThermoFisher).

**
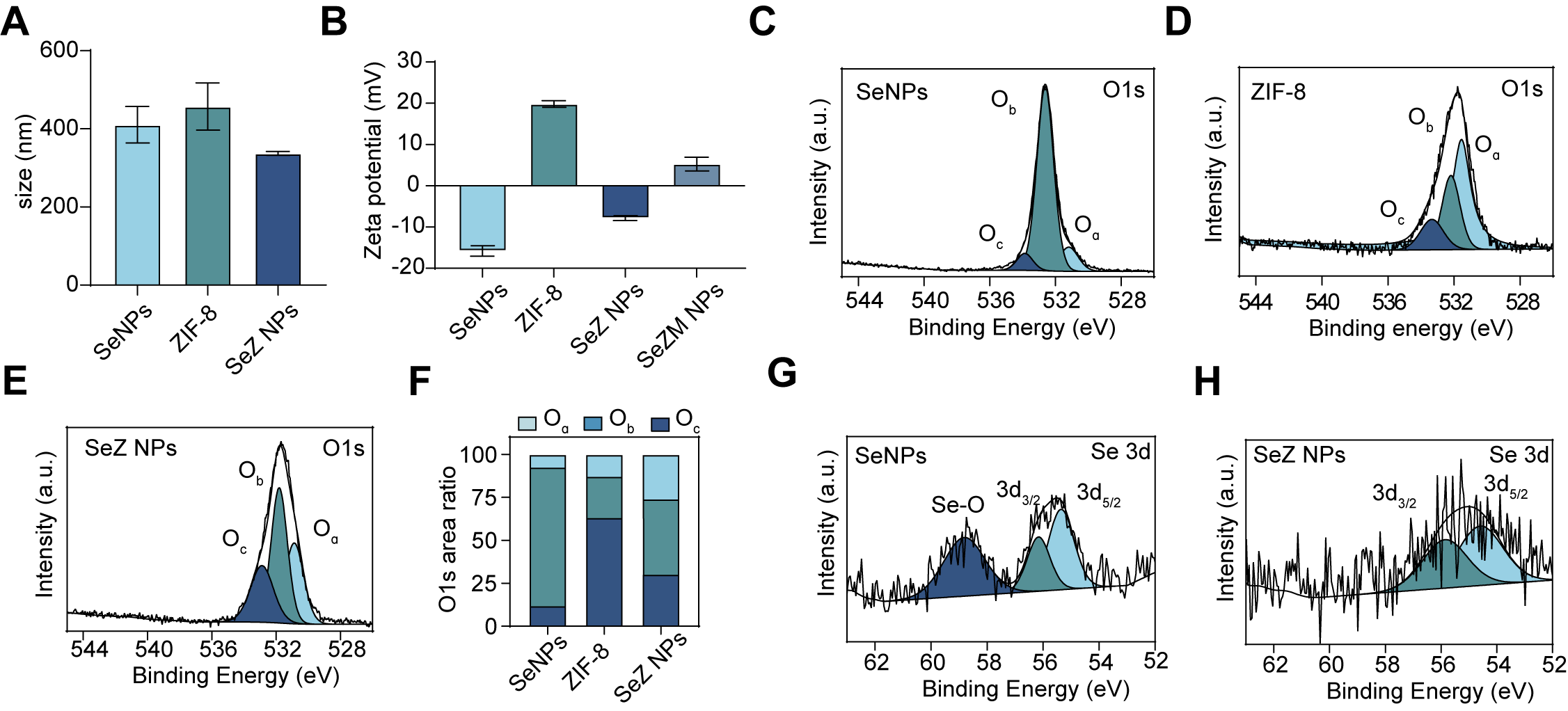
**

**Figure S1. Physicochemical characterization of selenium nanoparticles.**

A) Hydrodynamic diameters of SeNPs, ZIF-8, and SeZ NPs.

B) Zeta potentials of SeNPs, ZIF-8, SeZ NPs, and SeZM NPs.

C-E) XPS spectra of the O 1s region of SeNPs (C), ZIF-8 (D), and SeZ NPs (E), with peak deconvolution into Oₐ, O_b_, and O_c_ components.

F) Area ratio of Oₐ, O_b_, and O_c_ peaks in SeNPs, ZIF-8, and SeZ NPs.

G-H) High-resolution XPS spectra of Se 3d in SeNPs (G) and SeZ NPs (H), with Se–O and spin–orbit doublet (3d₃/₂ and 3d₅/₂) peaks identified.

All measurements were repeated independently at least three times.


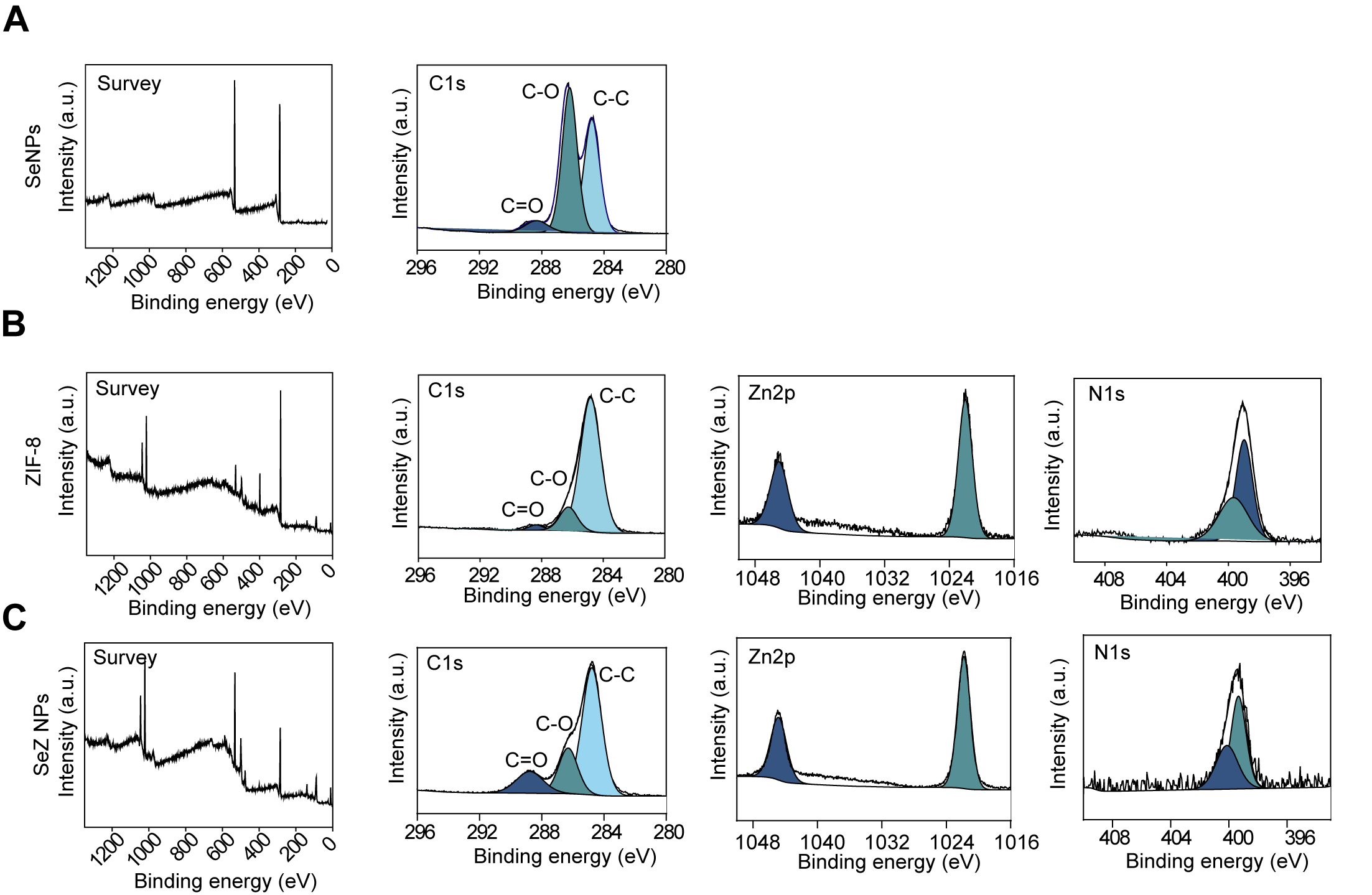


**Figure S2. XPS characterization of SeNPs, ZIF-8, and SeZ NPs.**

A) Survey and high-resolution C1s spectrum of SeNPs.

B) Survey and high-resolution spectra of ZIF-8, including C1s, Zn2p and N1s signals.

C) Survey and high-resolution spectra of SeZ NPs, including C1s, Zn2p and N1s signals.

Spectra are representative of three independent measurements.

**
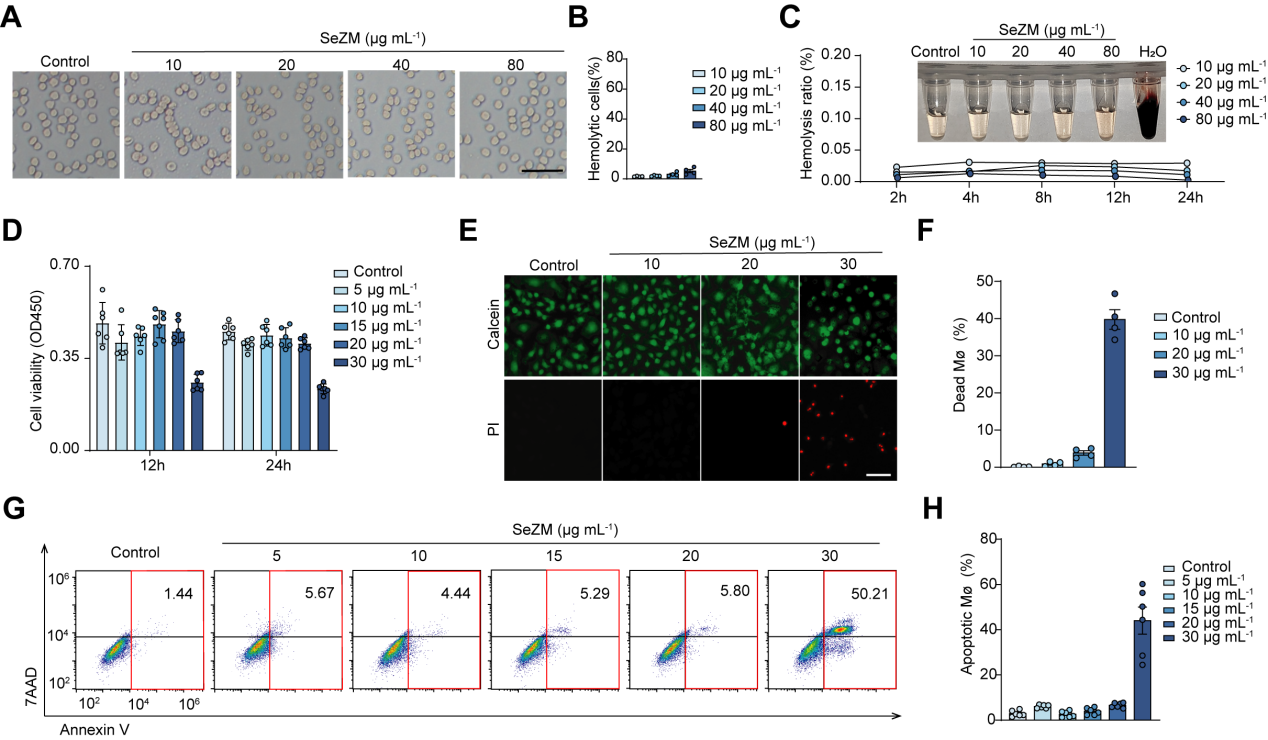
Figure S3. Evaluation the biocompatibility of SeZM NPs *in vitro*.**

A-B) Representative images (A) and quantification (B) of erythrocyte morphology after incubation with SeZM NPs (10, 20, 40, or 80 μg mL⁻¹) for 24 h. Scale bar: 50 μm. n = 4 per group.

C) Hemolysis ratio of erythrocytes treated with different concentrations of SeZM NPs at 2, 4, 8, 12 and 24 h. The image corresponds to the 24 h time point.

D) CCK-8 assay of BMDM viability after exposure to SeZM NPs for 12 and 24 h, measured by absorbance at 450 nm. n = 6 per group.

E-F) Representative Calcein/PI fluorescence images (E) and quantification of dead cells (F) in BMDMs treated with SeZM NPs for 24 h. Scale bar: 100 μm. n = 4 per group.

G-H) Representative flow cytometry plots (G) and quantification (H) of apoptotic BMDMs after 24 h SeZM NPs treatment, assessed by Annexin V/7AAD staining. n = 6 per group.


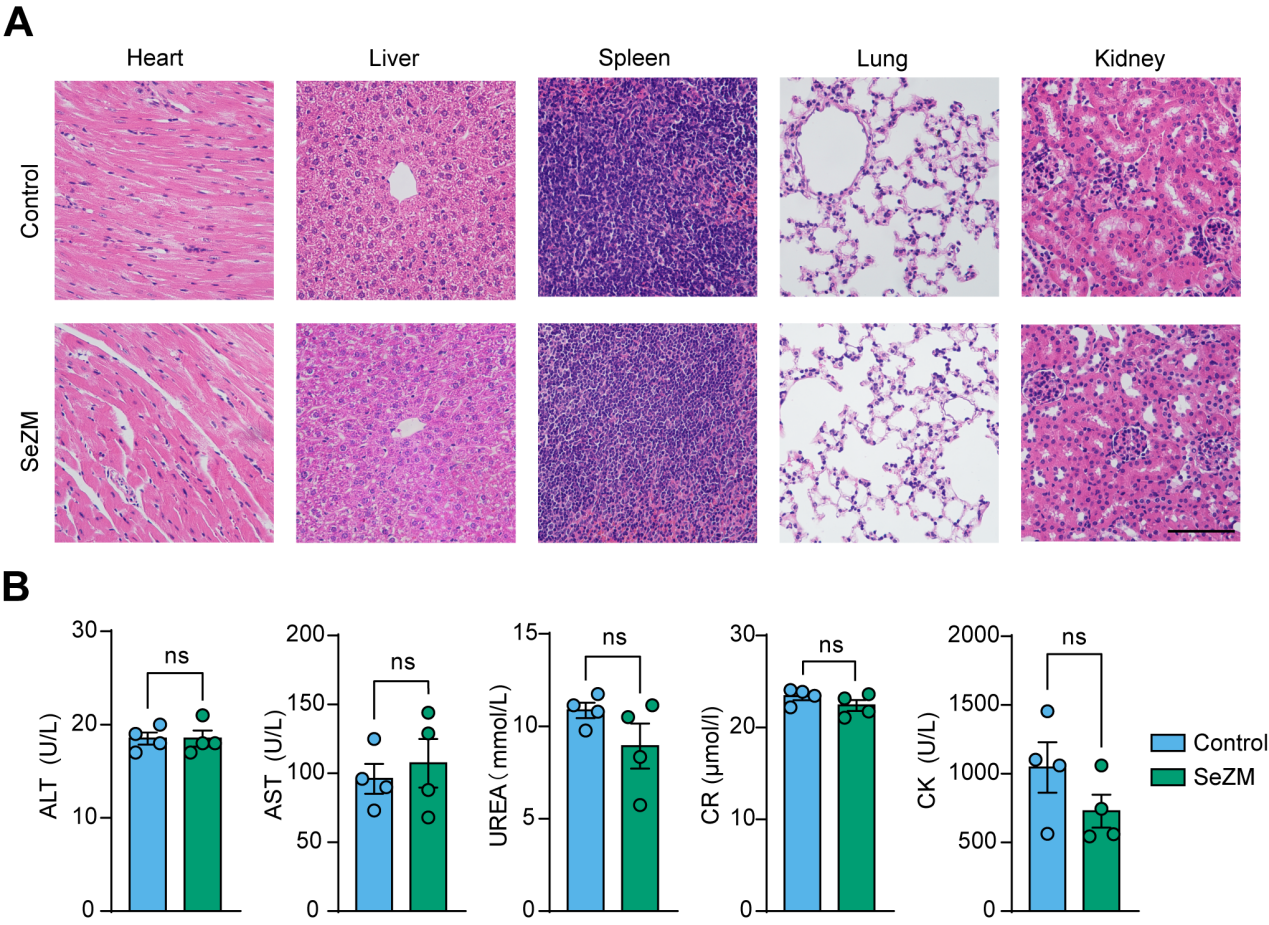


**Figure S4. Evaluation of the systemic toxicity of SeZM NPs in** **C57BL/6 mice.**
A) H&E staining images of heart, liver, spleen, lungs, and kidneys of **C57BL/6** mice five day after administration of PBS and SeZM NPs (10 mg kg ^-1^). Scale bar: 100 μm.

**B)** Serological results of the mice five days after administration of PBS and SeZM NPs (10 mg kg^-1^). The results included liver function markers (ALT, AST), renal function markers (UREA, CREA) and cardiac function markers (CK). n=4 per group. ns, not significant (unpaired two-tailed Student’s t-test).


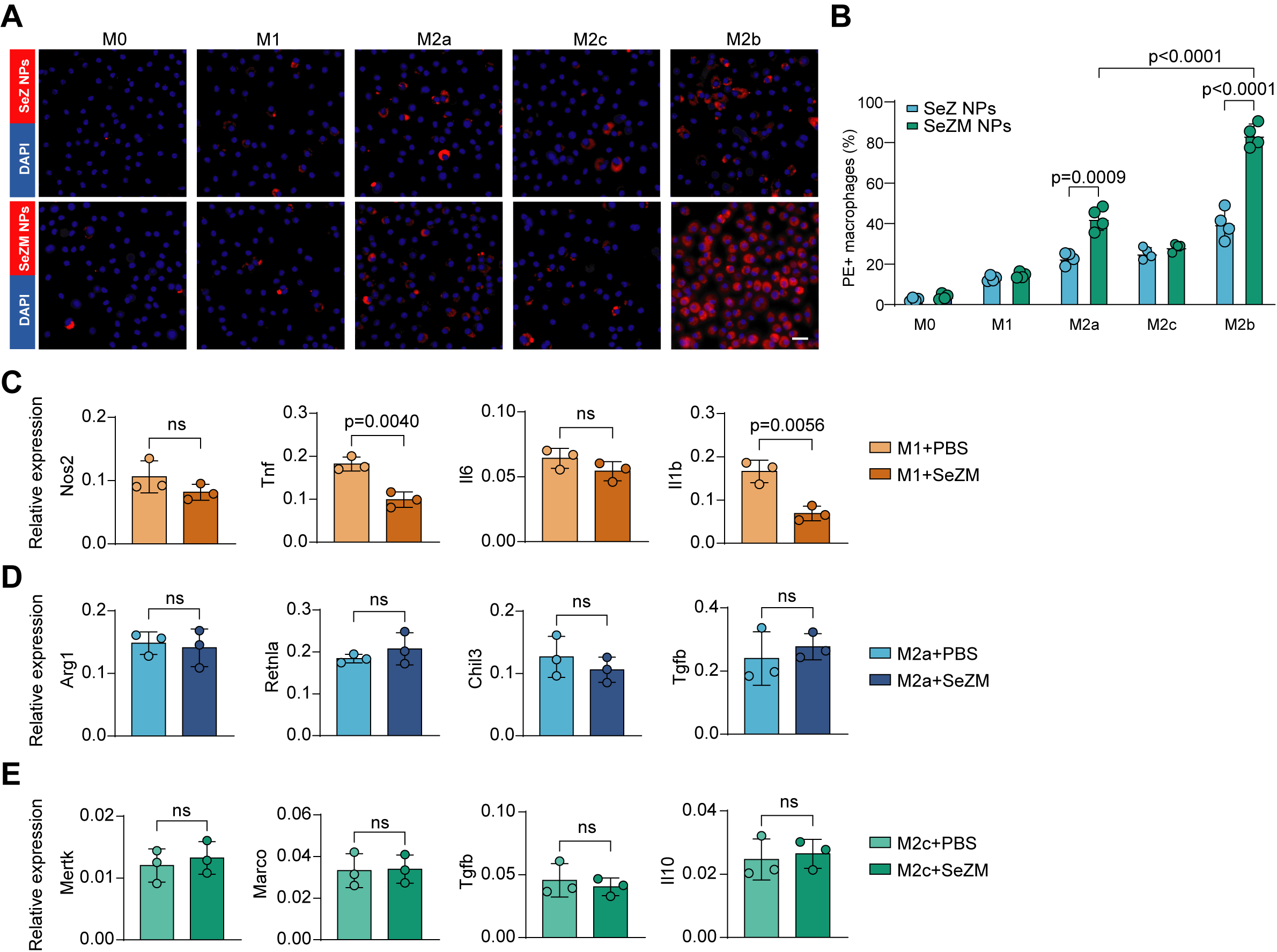


**Figure S5. Selective uptake of SeZM NPs by M2b macrophages *in vitro*.**

A) Representative fluorescent images of M0, M1, M2a, M2c and M2b macrophages after incubation with Rhodamine B-labeled SeZ or SeZM NPs for 3 h. Red: Rhodamine B; blue: DAPI. Scale bar: 10 μm.

B) Quantification of Rhodamine B positive (PE⁺) macrophages by flow cytometry after 3 h incubation with SeZ or SeZM NPs in each subpopulation. n = 4 per group.

C-E) qPCR analysis of polarization markers and inflammatory genes in M1 (C), M2a (D), and M2c (E) macrophages treated with PBS or SeZM NPs (10 μg mL⁻¹). n = 3 per group.

Data are shown as mean ± s.e.m. Statistical analysis was performed using two-tailed unpaired Student’s t-test.


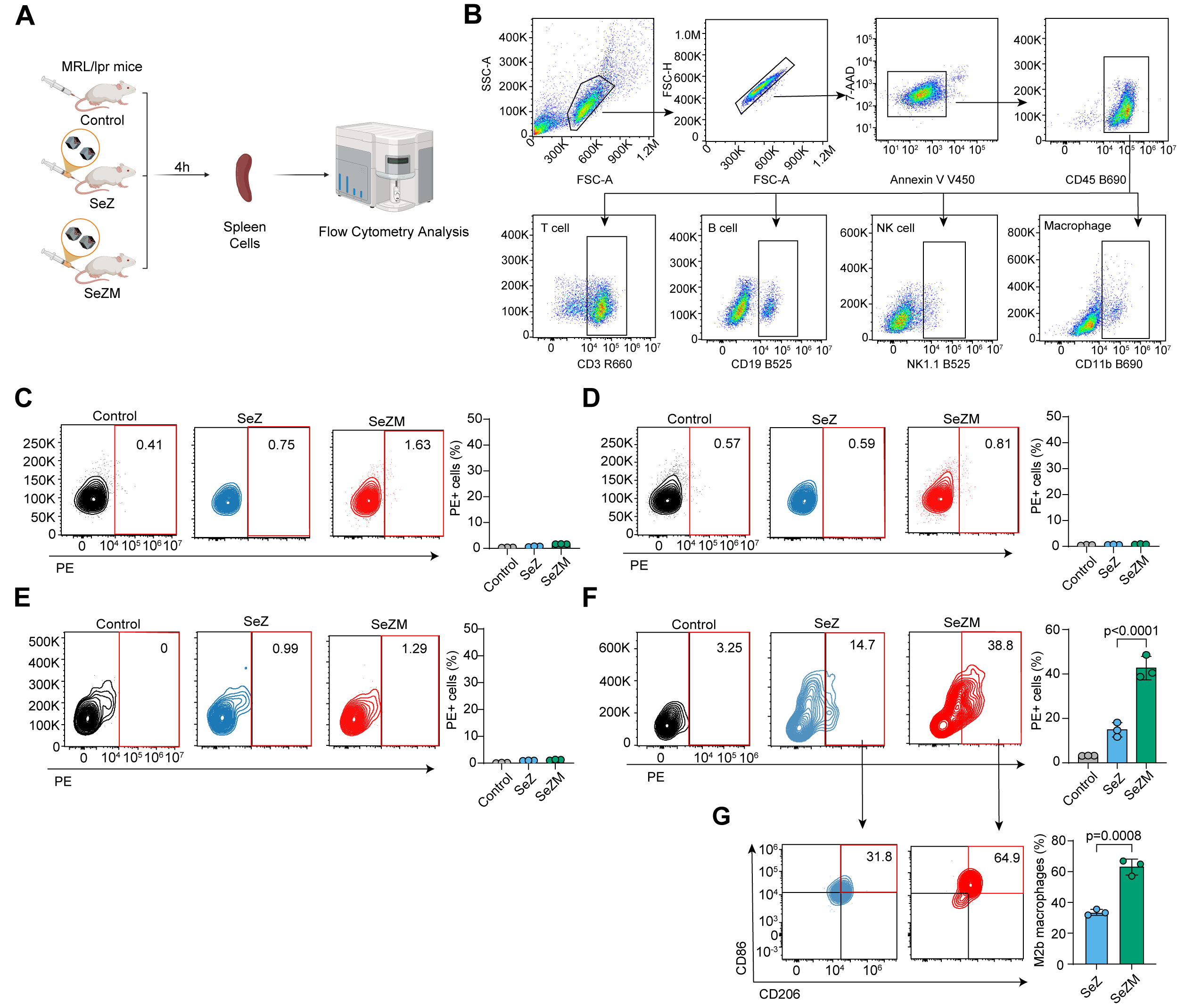


**Figure S6. Evaluation of the targeting efficiency of SeZM NPs in MRL/lpr lupus mice.**

A) Schematic flowchart of the experiment design. MRL/lpr mice were administrated intravenously with PBS (Control), Rhodamine B-labeled SeZ NPs (1 mg kg^-1^), or SeZM NPs (1 mg kg^-1^). Splenocytes were isolated 4 h after the administration. Flow cytometry analysis was performed to determine the Rhodamine B (PE^+^) signals in different immune cells.

B) Gating strategy of flow cytometry for T cells (CD3^+^CD45^+^), B cells (CD19^+^CD45^+^), NK cells (NK1.1^+^CD45^+^), and macrophages (CD11b^+^CD45^+^).

C-F) Representative flow cytometry images and quantification results of Rhodamine B-positive (PE^+^) cells in T cells (C), B cells (D), NK cells (E), and macrophages (F) from three groups.

G) Representative flow cytometry images and quantification results of CD86^+^CD206^+^ (MRC1^+^) M2b macrophages in Rhodamine B-positive (PE^+^) macrophages from mice treated with SeZ and SeZM NPs.


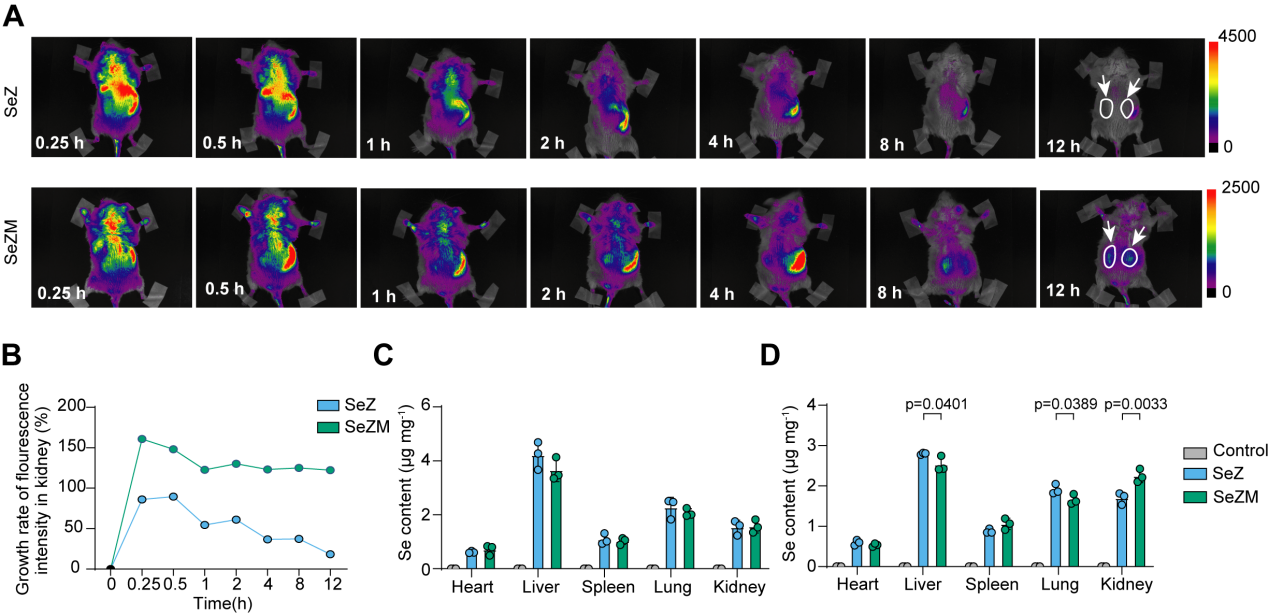


**Figure S7. Biodistribution of SeZ and SeZM NPs in MRL/lpr mice.**

A-B) Representative images (A) and quantification results (B) of the biodistribution of SeZ (2 mg kg^-1^) and SeZM (2 mg kg^-1^) NPs in MRL/lpr mice within 12 h after the administration.

C-D) Determination of selenium concentrations in different organs by ICP-MS at 4 h (C) and 12 h (D) after the administration of PBS (Control), SeZ or SeZM NPs in MRL/lpr mice. n=3 per group.

P values were determined using one-way ANOVA followed by Tukey’s *post hoc* test.


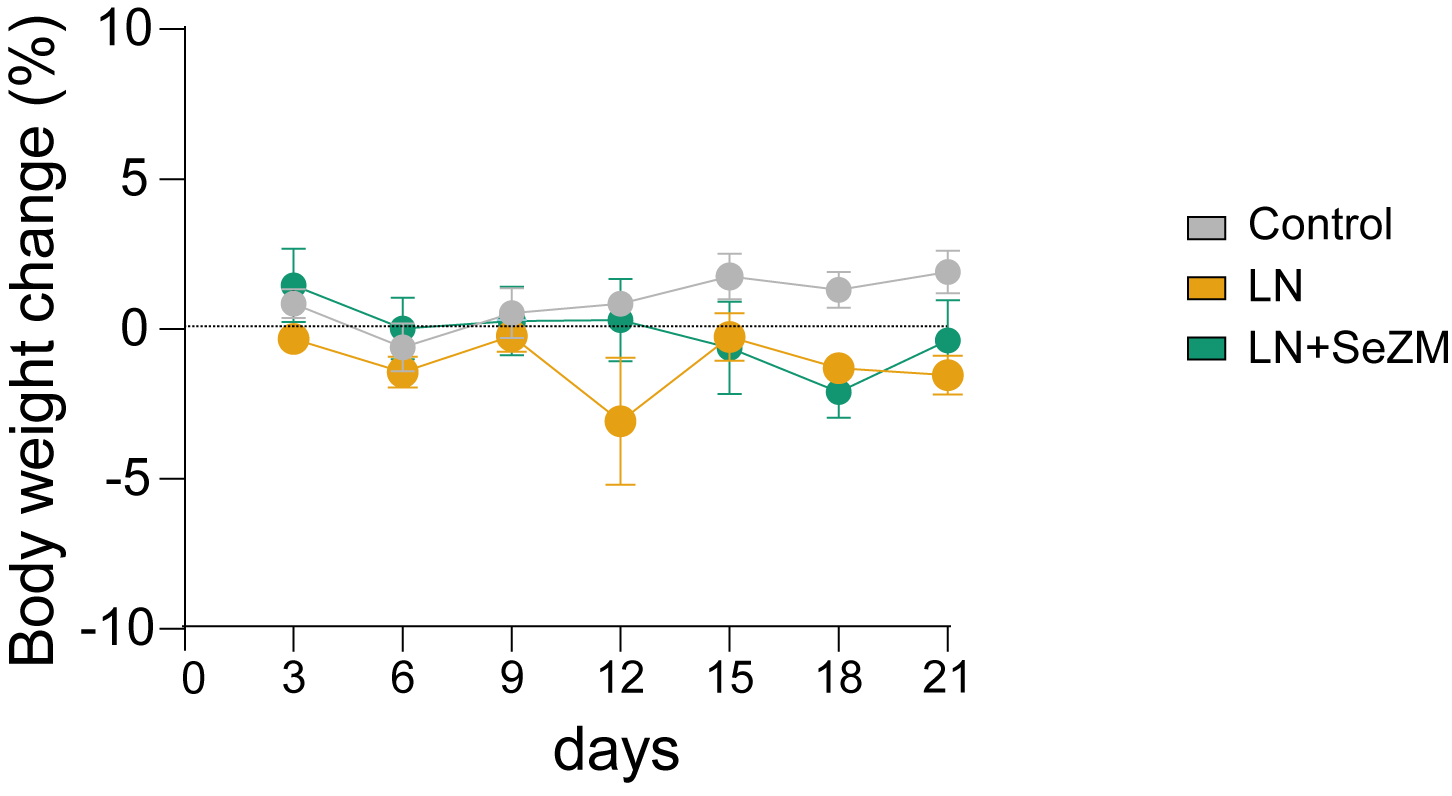


**Figure S8. Comparison of body weight changes from three groups.**Body weights were monitored throughout the 3-week treatment period in MRL/MpJ mice administered with PBS (Control), MRL/lpr mice treated with PBS (LN), or SeZM NPs (LN+SeZM). n = 5 per group.

**Table S1.** The primer sequences of the genes for RT-PCR.
