# Supplementary material for: Reprogramming M2b Macrophages via GPX1 Activation by Selenium Nanoparticles Attenuates Lupus Nephritis
Source: Adv Sci (Weinh). 2025 Dec 17;13(12):e19981. doi: 10.1002/advs.202519981 (PMC12948211; doi:10.1002/advs.202519981)
Supplement: Supplementary file 2 — Supporting Information [file ADVS-13-e19981-s002.pdf]

Table S1. The primer sequences of the genes for RT-PCR.

Species	Gene name	Primer direction	Primer pair
mouse	Tnf	forward 5'→3'	GGTGCCTATGTCTCAGCCTCTT
		reverse 5'→3'	GCCATAGAACTGATGAGAGGGAG
	Il6	forward 5'→3'	TCTATACCACTTCACAAGTCGGA
		reverse 5'→3'	GAATTGCCATTGCACAACTCTTT
	Il1b	forward 5'→3'	GAAATGCCACCTTTTGACAGTG
		reverse 5'→3'	TGGATGCTCTCATCAGGACAG
	Cias1	forward 5'→3'	TCACAACTCGCCCAAGGAGGAA
		reverse 5'→3'	AAGAGACCACGGCAGAAGCTAG
	Nos2	forward 5'→3'	GAGACAGGGAAGTCTGAAGCAC
		reverse 5'→3'	CCAGCAGTAGTTGCTCCTCTTC
	Cxcl9	forward 5'→3'	CCTAGTGATAAGGAATGCACGATG
		reverse 5'→3'	CTAGGCAGGTTTGATCTCCGTTC
	Cxcl10	forward 5'→3'	ATCATCCCTGCGAGCCTATCCT
		reverse 5'→3'	GACCTTTTTTGGCTAAACGCTTTC
	Gpx1	forward 5'→3'	CGCTCTTTACCTTCCTGCGGAA
		reverse 5'→3'	AGTTCCAGGCAATGTCGTTGCG
	Gpx3	forward 5'→3'	ATCCTGCCTTCTGTCCCTGCTC
		reverse 5'→3'	TGGTGAGGGCTCCATACTCGTA
	Selenoh	forward 5'→3'	AGCTACCTGTGCAAGTGAACC
		reverse 5'→3'	GGCCCTTCTTAATACCAGTCCAG
	Selenow	forward 5'→3'	TGAGGAGGTGCTGAAAGTGA
		reverse 5'→3'	CTGCTTTCTGCTTGTCAGGA
	Csf2	forward 5'→3'	AACCTCCTGGATGACATGCCTG
		reverse 5'→3'	AAATTGCCCCGTAGACCCTGCT
	Il23a	forward 5'→3'	CATGCTAGCCTGGAACGCACAT
		reverse 5'→3'	ACTGGCTGTTGTCCTTGAGTCC
	Il12b	forward 5'→3'	TTGAACTGGCGTTGGAAGCACG
		reverse 5'→3'	CCACCTGTGAGTTCTTCAAAGGC

Species	Gene name	Primer direction	Primer pair
mouse	Selenot	forward 5'→3'	TTTCGGCATGCAAGCTCCTAGC
		reverse 5'→3'	CACCTGTTGACATACACTGGTTC
	Selenop	forward 5'→3'	CCCCGGAGTGGTACATAGGAG
		reverse 5'→3'	CAGGTCTTCCAATCTGGATGC
	Txnrd3	forward 5'→3'	CCGTCCTTCTTCGTGGCTTTGA
		reverse 5'→3'	GGTGGACTTAGCCACGACTTTC
	IL23a	forward 5'→3'	CATGCTAGCCTGGAACGCACAT
		reverse 5'→3'	ACTGGCTGTTGTCCTTGAGTCC
	Ifng	forward 5'→3'	CAGCAACAGCAAGGCGAAAAAGG
		reverse 5'→3'	TTTCCGCTTCCTGAGGCTGGAT
	Il18	forward 5'→3'	GACAGCCTGTGTTCGAGGATATG
		reverse 5'→3'	TGTTCTTACAGGAGAGGGTAGAC
	Il17	forward 5'→3'	CAGACTACCTCAACCGTTCCAC
		reverse 5'→3'	TCCAGCTTTCCCTCCGCATTGA
	Ccl2	forward 5'→3'	GCTACAAGAGGATCACCAGCAG
		reverse 5'→3'	GTCTGGACCCATTCCTTCTTGG
	Gapdh	forward 5'→3'	CATCACTGCCACCCAGAAGACTG
		reverse 5'→3'	ATGCCAGTGAGCTTCCCGTTCAG
